# Targeted Hyaluronan Degradation Enhanced Tumor Growth Inhibition in Gastrointestinal Cancer Models

**DOI:** 10.3390/cancers17213411

**Published:** 2025-10-23

**Authors:** Fulai Zhou, Guangmao Mu, Honglei Bi, Limin Chen, Zhengxia Zha, Ying Jin, Mark L. Chiu

**Affiliations:** 1 Research & Development Department, Tavotek Biotherapeutics, Suzhou 215000, China; 2 Research & Development, Tavotek Biotherapeutics, Spring House, PA 19102, USA

**Keywords:** stromal hyaluronan, FAP, LRRC15, hyaluronidase, solid tumor microenvironment

## Abstract

**Background.** The dense hyaluronan (HA)-rich stroma in solid tumors can prevent effective tumor growth inhibition by hindering drug delivery and immune cell infiltration. However, the degradation of HA alone by systemic delivery of hyaluronidase has not shown significant improvement of tumor growth inhibition. **Objectives/Methods.** In this study, we targeted hyaluronan degradation by using antibody–enzyme (AbEn) molecules by fusing antibodies to a recombinant human hyaluronidase (HYAL). **Results.** The AbEn molecules were stable, retained both antigen-binding and enzymatic activities, and demonstrated a prolonged serum half-life of 132 h in rodent models. In the HA-rich colorectal cancer model, the cancer-associated fibroblast (CAF)-directed AbEn, TAVO423 (FAP × LRRC15 × HYAL trispecific antibody) achieved greater intratumoral HA depletion resulting in superior tumor growth inhibition compared to untargeted HYAL. Furthermore, the combination of TAVO423 in combination with other solid tumor cell targeting modalities such as 5-fluorouracil (5-FU), anti-PD-L1 monoclonal antibody, a PD-L1 × CD3 bispecific T-cell engager (TCE), and a CD318-targeting antibody–drug conjugate (ADC) all demonstrated enhanced tumor growth inhibition (TGI) values of 49–67% as compared to the respective monotherapy TGI values of 1–28%. In addition, TAVO423 improved the antitumor response of a 5T4 × CD3 TCE with an increase in TGI from 73% to 92% in an in vivo HA-rich pancreatic cancer model. The CAF-targeted HA degradation mediated by TAVO423 also reversed immune exclusion by increasing the density of CD8^+^ tumor-infiltrating lymphocytes (TILs) by 6–9-fold and synergized with PD-1 blockade to enhance TGI from 33% to 51% in an in vivo immunocompetent EMT-6 breast cancer model. **Conclusions.** These findings demonstrated the broad potential of the modular AbEn platform for targeted HA degradation to overcome barrier entry in stromal HA-rich solid tumors.

## Simple Summary

Stronger tumor growth inhibition was demonstrated with targeted disruption of the hyaluronan (HA) barrier that surrounded solid tumor cells. Systemic delivery of hyaluronidase can break down HA but is not effective when used alone and can cause adverse effects. We demonstrated how an “antibody–enzyme complex” (AbEn) disrupted the HA barrier via homing to the tumor or cancer associated fibroblast cells. In mouse models of colorectal cancer, these targeted AbEn molecules inhibited tumor growth better and enhanced the effectiveness of chemotherapy, immunotherapy, and other cancer drugs. Likewise, the AbEn molecules allowed the immune cells to have stronger anti-tumor growth activity.

## 1. Introduction

Tumor cells in the solid tumor is a part of a highly complex tumor microenvironment (TME), which includes the desmoplastic stroma, immune cells, blood vessels, cytokines, and other non-cellular components [1,2]. Each of these components participate in interactions that modulate tumor metabolism, growth, metastasis, immune evasion, as well as resistance to drug therapy [3]. In recent decades, anti-cancer therapies, such as targeted tumor cell therapies and immune checkpoint inhibitors (ICI), have emerged as effective treatments for certain solid tumors [4]. Unfortunately, many patients either do not respond to such therapies or experience relapses after having initial responses; concomitantly, the tumor cell resistance mechanisms remain poorly understood [5,6,7]. We hypothesized that better tumor control in the TME required control of the tumor cells and the stroma, a major component of the TME. The TME stroma includes the cancer-associated fibroblasts (CAFs) and the extracellular matrix (ECM) that act as physical barriers that hinder effective drug delivery and immune cell infiltration to make contact with the tumor cells. Additionally, the stroma can promote tumorigenesis, cancer progression, metastasis, and therapy resistance, thereby limiting the effectiveness of treatments for solid tumors [8]. We presented how disruption of these TME barriers could improve therapeutic outcomes.

CAFs are key elements of the tumor stroma that promote tumor growth, invasion, and metastasis [9]. The development of the CAF phenotype is associated with various CAF-related markers, most notably the increased levels of the fibroblast activation protein (FAP). FAP has been considered a promising therapeutic target since FAP positive (FAP^+)^ CAFs are linked to immunosuppressive tumor microenvironments and correlate with worse prognoses in solid tumor cancers [10,11]. Various strategies targeting FAP, including small molecule inhibitors, monoclonal antibodies (mAbs), antibody–drug conjugates (ADCs), and chimeric antigen receptor T (CAR-T) cells, have demonstrated therapeutic effects in murine tumor models [12,13,14]. Another dominant CAF marker is the leucine-rich repeat-containing protein 15 (LRRC15), which has minimal expression in normal tissues. Elevated LRRC15 in stromal fibroblasts of multiple solid tumors, osteosarcoma, soft tissue sarcomas, and mesenchymal cancer cells are associated with higher tumor grades and worse outcomes in osteosarcoma and soft tissue sarcomas [15,16]. LRRC15^+^ CAFs also promote tumor growth and suppress CD8^+^ T cell function, indicating that therapies targeting LRRC15 may enhance patient survival and responses to immunotherapy [17]. Nonetheless, FAP and LRRC15-targeted therapies that focus on CAFs alone may not be sufficient for cancer therapy. Rather, additional mechanism of action strategies to dismantle the tumor stromal barriers could result in better tumor growth inhibition. Combining CAF-targeted therapies with agents that target the ECM could allow more effective inhibition of tumor cells in the tumor microenvironment.

Hyaluronic acid (HA) or hyaluronan is the major polysaccharide component of the ECM [18]. Beyond serving as a barrier, HA polymers in the ECM of solid tumors regulate cellular functions in a molecular-weight (MW)-dependent manner through interactions with HA receptors, particularly cluster of differentiation 44 (CD44) and the hyaluronan-mediated motility receptor (RHAMM) [19]. High-molecular-weight (HMW) HA exhibits anticancer effects by inhibiting cell proliferation and angiogenesis, while low-molecular-weight (LMW) HA promotes tumorigenesis [20,21]. The conversion of HMW-HA to LMW-HA is facilitated by hyaluronidase (HYAL) enzymes, including HYAL1, HYAL2, HYAL3, and PH20 (sperm adhesion molecule 1) [22]. PH20, the only neutral-active hyaluronidase, is commonly employed to hydrolyze HA in tumors or is co-administered with subcutaneously injected mAbs to enhance dispersion and absorption [23]. Since, PH20 HYAL has a short half-life, a pegylated recombinant human PH20 variant (PEGPH20) can remodel the stroma of HA-rich tumors by improving the intra-tumor distribution of anticancer drugs and enhancing their therapeutic efficacy without increasing toxicity [24,25,26]. However, despite promising results in phase I and II studies for tumors with high HA expression, the phase III study of PEGPH20 combined with chemotherapy for pancreatic cancer failed to extend patient survival [27,28,29,30]. Perhaps, the systemic delivery of PEGPH20 for HA depletion alone may not be sufficient for effective treatment.

To address these challenges, we hypothesized that a targeted hyaluronidase delivery platform that specifically degraded stromal HA within the TME could enhance treatment efficiency as compared to non-targeted HA depletion approaches. We also showed how targeted CAF delivery using two well-known CAF targets: FAP and LRRC15 [17,31]. TAVO423 is a FAP × LRRC15 × HYAL trispecific AbEn that effectively degraded HA, delayed tumor growth, and enhanced the efficacy of various therapeutics in an in vivo HA-rich colorectal cancer model. Furthermore, combining CAF-targeted HA degradation with PD-1/PD-L1 blockade significantly increased tumor-infiltrating T cells in a syngeneic breast cancer model, thereby amplifying the antitumor responses to immune checkpoint therapy. We demonstrated how CAF-targeted hyaluronidase could improve treatment outcomes in HA-rich solid tumors.

## 2. Materials and Methods

### 2.1. Cell Lines

Human Expi293F cells (Gibco, #A14527) were cultured in suspension using Expi293 expression medium (Gibco, #A1435101, Grand Island, NY, USA), while ExpiCHO-S cells (Gibco, #A29127) were cultured in suspension with ExpiCHO expression medium (Gibco, #A2910001). Both cell types were routinely cultured at a density of 3–5 × 10^6^ cells per mL to maintain a healthy state, under conditions of 8% CO_2_, ≥80% relative humidity, and a shaking speed of 125 rpm in a 37 °C incubator. Human RKO (#TCHu116) and HCC827 (#SCSP-538) cells were obtained from the National Collection of Authenticated Cell Cultures of the Chinese Academy of Sciences (Shanghai, China), while human U-87 MG (#HTB-14) cells were purchased from the American Type Culture Collection (ATCC, Manassas, VA, USA). RKO cells were cultured in Dulbecco’s modified Eagle’s medium (DMEM, Gibco, #10566016) supplemented with 10% (*v*/*v*) fetal bovine serum (FBS, Sigma, #F8318, St. Louis, MO, USA). HCC827 cells were cultured in RPMI-1640 medium (Corning, #10-040-CVRC, Corning, NY, USA) with 10% FBS, and U-87 MG cells were cultured in Eagle’s Minimum Essential Medium (EMEM, Corning, #10-009-CV) with 10% (*v*/*v*) FBS. BxPC-3-WT (ATCC, CRL-1573) and BxPC-3-HAS3 stable cell lines (GeneChembio, Shanghai, China) were cultured in DMEM (Gibco, #10566016). All cancer cell lines were incubated in a humidified chamber with 5% CO2 at 37 °C. The EMT-6 cells (ATCC) were cultured in vitro using Waymouth’s MB752/1 medium (Gibco) supplemented with 15% (*v*/*v*) FBS at 37 °C in a 5% CO_2_ atmosphere. Cells in exponential growth phase were harvested, counted with a cell counter, and then used for tumor inoculation.

### 2.2. Constructs, Expression, and Purification

All codon-optimized genes were synthesized and cloned into the pcDNA3.4 vector (GenScript, Nanjing, China). To enhance protein expression and secretion, a signal peptide (MAWVWTLLFLMAAAQSIQA) was inserted at the N-termini of each DNA open reading frame. For purification purposes, the C-terminus of the human PH20 sequence (L36-Y482) was linked to a histidine 6 (His6) tag or to an IgG1 Fc domain via a flexible polypeptide linker (GGGGS). The antibody amino acid sequences were annotated according to the Kabat numbering scheme [32]. The antibodies used in this study were expressed in the Expi293F system using the Expifectamine293 transfection kit (Gibco, #A14527), in accordance with the manufacturer’s recommendations. For the exemplary IgG1 monoclonal antibodies (mAbs), the transfection weight ratio of heavy chain (HC) to light chain (LC) plasmids was 2:3. The PH20-His6 and PH20-Fc proteins were expressed in ExpiCHO-S cells using electroporation (Etta Biotech, X-Porator F1, Suzhou, China ), following the manufacturer’s instructions. Six days post-transfection, the supernatants containing recombinant proteins and antibodies were harvested by centrifugation and filtration using a 0.2 μm filter.

For the purification of antibodies and PH20-Fc, the filtered supernatants were loaded onto a MabSelect SuRe column (Cytiva, #11003494, Marlborough, MA, USA) using an AKTA pure 25 system (Cytiva, #29018224). The column was washed with PBS, and the bound antibodies were eluted using an elution buffer containing 150 mM sodium citrate, pH 3.4 (prepared in-house) and collected in tubes with 1 M tris-HCl solution, pH 9.0 (Sangon Biotech, #8548128, Shanghai, China) for rapid neutralization. For the purification of PH20-His6 protein, the supernatant was incubated with TALON Metal Affinity Resin (Clontech, #635501, Mountain View, CA, USA) for 2 h at 4 °C. The resin was then packed and washed with 30 column volumes of wash buffer (PBS) before the protein was eluted in 150 mM imidazole.

The integrity and components of the purified protein were analyzed using sodium dodecyl sulfate polyacrylamide gel electrophoresis (SDS-PAGE) on precast SurePAGE 4–12% Bis-Tris gels (GenScript, #M00654) under reducing or non-reducing conditions, following the manufacturer’s instructions, and stained with Coomassie brilliant blue (prepared in-house). Monomeric purity was assessed using high-performance liquid chromatography-size exclusion chromatography (HPLC-SEC) on an Agilent 1260 Infinity II LC system (Agilent Technologies, #G7167A, Santa Clara, CA, USA) equipped with an AdvanceBio SEC column (Agilent Technologies, #PL1180-5301). The running buffer consisted of 150 mM KH_2_PO_4_ (Sigma, #104873), pH 7.3, at room temperature. Proteins containing aggregates or fragments were concentrated using an Amicon Ultra Centrifugal Filter (MWCO: 30 kDa, Millipore, #UFC5030, Billerica, MA, USA) and further purified via a pre-equilibrated Superdex 200 Increase 10/300 GL 24 mL column (Cytiva, #28990944). Peak fractions were collected for subsequent characterization.

The AbEn molecules were generated by mixing equimolar solutions of the HYAL-Fc with the parental targeting arms in the presence of 2-MEA using the controlled Fab-arm exchange (cFAE) technique [33]. Briefly, equimolar solutions of HYAL-Fc and the parental antibody at concentrations of 1–10 mg/L were mixed at a 1:1 molar ratio. Reduction was initiated by adding 2-mercaptoethylamine (2-MEA) to a final concentration of 75 mM, followed by incubation for 5 h at 31 °C. The 2-MEA was then removed by dialysis against a large volume of PBS for 16–18 h at 4 °C to facilitate re-oxidation and bispecific molecule formation. The formation of the antibody–enzyme conjugates were confirmed using several chromatographic methods. HPLC-SEC analysis verified successful bispecific AbEn formation based on the distinct molecular weight differences between the parental molecules (HYAL-Fc or the parental antibody) and the medium conjugate. The bispecific AbEn exhibited an average molecular weight consistent with the sum of its two parental molecules. Monomeric and monodisperse purity were assessed using an Agilent 1260 Infinity II LC system equipped with an AdvanceBio SEC column. An isocratic method was employed with a 150 mM KH_2_PO_4_ buffer (pH 7.3) at room temperature and detection at 280 nm. The exchange efficiency was confirmed to be over 90%, as calculated by the monomeric peak area expressed as a percentage of the total injection area. The amino acid numbering followed the EU index for the Fc domain mutations.

### 2.3. Hyaluronidase Activity Assay

The activity of the hyaluronidase enzyme was measured using a 45 min turbidimetric assay, as previously described [34]. Briefly, enzymes were first diluted to 2–5 units per mL in a buffer containing 20 mM sodium phosphate (pH 7.0), 77 mM sodium chloride, and 0.01% (*w*/*v*) bovine serum albumin, and were equilibrated at 37 °C for 10 min. The enzyme dilution was then immediately mixed with 0.3 mg/mL HA (Sigma-Aldrich, #H5388, St. Louis, MO, USA), which was prepared in a buffer containing 300 mM sodium phosphate (pH 5.35) at 37 °C. The mixture was swirled and incubated at 37 °C for exactly 45 min to monitor HA degradation. Following incubation, each test and blank sample was mixed promptly with an acidic albumin solution consisting of 24 mM sodium acetate, 79 mM acetic acid, and 0.1% (*w*/*v*) bovine serum albumin (pH 3.75 at 25 °C). After mixing and waiting for an additional 10 min at room temperature, the absorbance values at 600 nm were recorded. One unit of hyaluronidase activity was defined as a change in absorbance (A600) of 0.330 per minute at pH 5.35 and 37 °C in a 2.0 mL reaction mixture. The units of activity were determined according to a standard curve of hyaluronidase activity generated from a solution of bovine testicular hyaluronidase (Sigma). The standard curve relating absorbance to enzyme activity units (0–6 units of enzyme per mL of working solution) was fit by linear regression in Prism 8 (GraphPad, Version 8.0.2, San Diego, CA, USA).

### 2.4. Flow Cytometry

RKO or HCC827 cells were digested with enzyme-free cell dissociation buffer (Gibco, #WHO621 K121) and washed twice with precooled buffer (PBS supplemented with 2% (*v*/*v*) FBS). A total of 1 × 10^5^ cells and test articles at varying concentrations (starting from 33 nM with a series of 3-fold dilutions) were seeded into each well of a 96-well round-bottom plate and incubated on ice for 1 h to facilitate binding. After incubation, the cells were washed twice and stained with an Alexa Fluor 647 AffiniPure Goat Anti-Human IgG, Fcγ fragment-specific detection antibody (dilution 1:5000, Jackson ImmunoResearch, #109-605-190) at 4 °C for 30 min in the dark. Following this, two additional washing steps were performed, and the cells were resuspended for flow cytometric analysis on a CYTOFLEX flow cytometer (Beckman Coulter, Miami, FL, USA) using CytExpert 2.4.0.28 software. The EC50 and Emax values were calculated for each curve fitted using a four-parameter logistic equation in Prism 8 (GraphPad, Version 8.0.2).

### 2.5. T-Cell Cytotoxicity Assay

The T-cell-mediated cytotoxicity of PD-L1 × CD3 bispecific antibodies was assessed using PD-L1-expressing cancer cell line RKO and PBMCs. Briefly, target cells were collected and seeded into 96-well round-bottom plates at a density of 20,000 cells per well. Human PBMCs, obtained from healthy donors (SAILYBIO, #SC12282W), were rapidly thawed in a 37 °C water bath and added to the target cells at a 5:1 effector-to-target (E:T) cell ratio. Bispecific antibodies, at varying concentrations (starting at 10 nM and serially diluted 3-fold across the plate to generate an 8-point titration curve), were added to each well containing the target cells. The plates were then incubated under standard cell culture conditions for 72 h to allow cytotoxicity to occur. Control wells included cells alone, cells with PBMCs, and cells treated with 0.2% (*w*/*v*) Triton-X-100 (total lysis, Sigma, #T8787, St. Louis, MO, USA) to establish baseline values for calculating the percentage of killing. After incubation, 100 μL of supernatant from each well was transferred to a new flat-bottom 96-well plate for lactate dehydrogenase (LDH) detection (Roche, #11644793001), following the manufacturer’s protocol.

### 2.6. In Vitro Cell Viability Assay

RKO cells were seeded into 96-well plates at 5000 cells/well. After overnight incubation, a serially diluted solution of CD318-MMAE ADC was added. Cell viability was evaluated after 4 days using the CellTiter-Glo^®^ Luminescent Cell Viability kit (Promega, Madison, WI, USA) according to the manufacturer’s instructions. The final luminescence signal was measured using a multiplate reader (Tecan). Concentration–response curves and half-maximal inhibitory concentration (IC_50_) values were generated by nonlinear regression using a sigmoidal curve fit with variable slope in GraphPad Prism 8.0. Assays were performed in triplicate for each data point.

### 2.7. ELISA

A standard ELISA protocol was employed to measure the levels of FAP and LRRC15 antigen binding. Briefly, a 96-well microplate was coated with the target antigen diluted in coating buffer (1 µg/mL) and incubated overnight at 4 °C. After washing with PBST (PBS containing 0.05% (*w*/*v*) Tween-20), the plate was blocked with 5% (*w*/*v*) BSA or non-fat dry milk in PBS for 1–2 h at room temperature to prevent non-specific binding. Serial dilutions of the primary antibody were then added to the wells and incubated for 1–2 h, followed by washing and incubation with an enzyme-conjugated secondary antibody for 1 h. After a final wash, the substrate (e.g., TMB) was added, and the reaction was stopped with 1 M H_2_SO_4_. Absorbance was measured at 450 nm using a Tecan microplate reader.

For serum HA fragment measurement, Balb/c nude mice (*n* = 3 per group; 6–10 weeks old; Nanjing GemPharmatech Co., Ltd. Nanjing, China) were subcutaneously inoculated with 3 × 10^6^ RKO tumor cells. Tumor-bearing mice were randomly grouped into treatment cohorts once mean tumors reached 200–250 mm^3^. Following a single intravenous injection of 1 mg/kg HYAL-Fc IgG1 fusion protein, blood samples (20 μL) were collected at 2, 8, 24, 48, and 72 h post-administration. The HA levels in mice serum at different time points were quantified using the Hyaluronan Valukine ELISA Kit (R&D Systems, # VAL152), according to the manufacturer’s instructions.

For intratumoral HA measurement, tumors from RKO xenograft models were harvested, weighed, and snap-frozen in liquid nitrogen. Frozen tissues were pulverized and homogenized on ice in a lysis buffer (100 mM Tris-HCl, pH 7.4, 1% (*w*/*v*) Triton X-100) supplemented with protease inhibitors. The homogenates were incubated at 4 °C for 2 h to extract HA, followed by centrifugation at 12,000× *g* to clarify the lysates. The concentration of HA in the supernatants was quantified using the Hyaluronan Quantikine ELISA Kit (DHYAL0, R&D Systems, Minneapolis, MN, USA) according to the manufacturer’s instructions. Sample lysates were diluted as necessary in the provided calibrator diluent to fall within the assay’s linear range. The intratumoral HA concentration was calculated from the standard curve of HA concentration with OD value, and normalized to the initial tumor weight, expressed as μg of HA per mg of tissue.

### 2.8. In Vivo Efficacy Studies

Mice were housed in a specific pathogen-free (SPF) facility in individual ventilated cage (5/cage) on corn cob bedding with crumpled tissue paper for enrichment. The SPF facility maintained a 12:12 light cycle (22 ± 1 °C, 60% humidity). Animals had ad libitum access to irradiated chow and autoclaved water. Health was monitored daily; no abnormalities were observed. For in vivo efficacy studies of the combination therapy involving TAVO423 with anti-PD-L1 mAb or PD-L1 × CD3 bispecific antibody in the RKO xenograft model or 5T4 x CD3 in the BxPC-3-HAS3 xenograft model, human PBMCs containing CD3-positive effector T cells were engrafted into immunocompromised mice five days prior to tumor cell inoculation. After human PBMC reconstitution, tumors were established by injecting 3 × 10^6^ cells into the flanks of 6–10 weeks old female Balb/c nude mice. In other efficacy studies using the RKO xenograft model, Balb/c nude female mice received subcutaneous injections of 3 × 10^6^ RKO tumor cells. Once tumors reached an average volume of 100–150 mm^3^, mice were randomized (*n* = 6 or 8) and treated with isotype controls or test agents at specified dosages and regimens. 5-Fluorouracil (5-FU, Shanghai Xudong Haipu Pharmaceutical Co., Ltd., # H31020593, Shanghai, China) and anti-PD-L1 mAb (Atezolizumab, MedChemExpress., # HY-P9904, Shanghai, China) were purchased from external suppliers. The CD318-MMAE ADC was prepared in-house via cysteine conjugation. Other antibodies used in the animal studies were also prepared in-house. The dose levels were selected based on prior in-house dose–response studies showing efficacy without toxicity. The primary outcome was tumor volume reduction. Tumor response was monitored twice weekly by measuring tumor size and mouse body weight. Tumor volumes were measured using calipers, with volumes calculated in mm^3^ using the formula: V = (L × W × W)/2, where V is tumor volume, L is tumor length (longest dimension), and W is tumor width (perpendicular to L). At the study endpoint, mice were sacrificed, and tumors were excised and weighed to assess tumor shrinkage. For humane endpoints, mice were euthanized if body weight loss >20% or individual mouse with tumor volume exceeding 3000 mm^3^. Daily monitoring included weight, tumor size, and activity. No severe adverse events (e.g., >20% weight loss) were observed in any group during the study. All procedures involving animal care, handling, and treatment complied with the guidelines of the Institutional Animal Care and Use Committee (IACUC) of Suzhou Charles River Accelerator and Development Lab (Approved Protocols: P202302160002-20240711 and P202302160002-20241219).

For the in vivo efficacy study of anti-mouse PD-1 antibody (anti-mouse PD-1 (CD279), BioXcell, #BP0146) in the syngeneic model, each mouse was subcutaneously inoculated in the right rear flank with 5 × 10^5^ EMT-6 cells of 6–10 weeks old female Balb/c mice (Shanghai Lingchang Biotechnology Company Limited, Shanghai, China) to promote tumor growth. Randomization was initiated once the mean tumor size reached 80–100 mm^3^. Mice (*n* = 5 per group) were then randomized and treated with either isotype controls or test agents at specified doses and regimens. The day of randomization was designated as day 0. Following tumor inoculation, animals were checked daily for morbidity and mortality. Routine monitoring included assessments of tumor growth effects and treatment-related impacts on behavior, such as mobility, food and water intake, body weight changes (measured two or three times per week post-randomization), eye and hair condition, and other abnormalities. Mortality and clinical signs were recorded in detail for each animal. At the end of the research, tumors were collected for IHC staining of tumor infiltrating lymphocytes. Tumor volumes were measured two or three times weekly using calipers, with volumes calculated in mm^3^ using the formula: V = (L × W × W)/2, where V is tumor volume, L is tumor length (longest dimension), and W is tumor width (perpendicular to L). All dosing, tumor measurements, and body weight assessments were performed inside a laminar flow cabinet to maintain standard conditions. The efficacy study with EMT-6 model was performed by Crown Bioscience (Taicang, China) according to the IACUC-approved protocol (AN-2304-07-1441) and in accordance with the regulations of the Association for Assessment and Accreditation of Laboratory Animal Care (AAALAC).

### 2.9. Pharmacokinetics

Pharmacokinetics (PK) study was conducted on non-tumor-bearing female BALB/c nude mice. Mice received an intravenous injection of 1 mg/kg of human IgG1 isotype or HYAL-Fc IgG1 fusion protein. Serial blood samples were collected at 2, 8, 24, 48, 120, 192, 240, and 360 h post the single dose. Blood samples were allowed to clot for 2 h at room temperature, then centrifuged at 10,000 rpm for 10 min. The serum was separated and stored at −80 °C until analysis. Serum concentrations of human IgG1 isotype and HYAL-Fc IgG1 fusion protein were measured using the human IgG precoated ELISA kit (Dakewe, #1128162, Shenzhen, China). The half-life value was estimated using the noncompartmental analysis module in WinNonlin^®^ (version 8.3; Certara, Mountain View, CA, USA).

### 2.10. Immunofluorescence Staining

IHC staining for FAP and LRRC15 was performed on formalin-fixed, paraffin-embedded (FFPE) sections from mouse xenograft samples using rabbit antibody clone (Solarbio, Cat# K110906P-50ul for FAP, and Cat# ab150376 for LRRC15, Beijing, China). HA staining was performed using Alcian Blue [35] or IHC with the HA-binding protein (Amsbio, Cat#AMS.HKD-BC41, Cambridge, MA, USA). Briefly, FFPE sections were deparaffinized in xylene and rehydrated through a graded ethanol series. Antigen retrieval was carried out using EDTA (pH 9.0) at 95–100 °C for 20 min. Endogenous peroxidase activity was quenched with 3% (*v*/*v*) hydrogen peroxide, and nonspecific binding was blocked with 5% (*w*/*v*) normal serum for 30 min. Sections were then incubated with primary antibodies (anti-mouse CD3, Abcam, #ab16669; anti-mouse CD8, Cell Signaling, #98941S; anti-mouse CD11b, Cell Signaling, #93169S; anti-mouse CD45, Cell Signaling, #70257S) overnight at 4 °C, followed by incubation with a horseradish peroxidase (HRP)-conjugated secondary antibody for 30 min at room temperature. Detection was performed using a DAB chromogen substrate, and sections were counterstained with hematoxylin, dehydrated, and mounted. Appropriate positive and negative controls were included to ensure staining specificity and accuracy. All images were acquired using the NanoZoomer-HT 2.0 Image system (Hamamatsu, Japan) at 40 times magnification (40×).

For image analysis for T cells infiltration in EMT-6 FFPE samples, all the images were analyzed with the HALO^TM^ platform. The whole slide image was analyzed, and big necrosis and stroma areas were excluded. Both the numbers of total cells and IHC positive cells were counted. IHC score was presented as the ratio of the positive cell counts against the total cell numbers of whole section.

### 2.11. Statistical Analyses

Statistical analyses were performed using GraphPad Prism (Version 8.0.2). The data were expressed as mean ± SEM values. For comparisons between two groups, an unpaired, two-tailed Student’s *t*-test was used. For comparisons across more than two groups, the one-way ANOVA was applied. A *p*-value of less than 0.05 was considered statistically significant.

## 3. Results

### 3.1. Engineering an Antibody–Enzyme Conjugate

**Figure 1:**
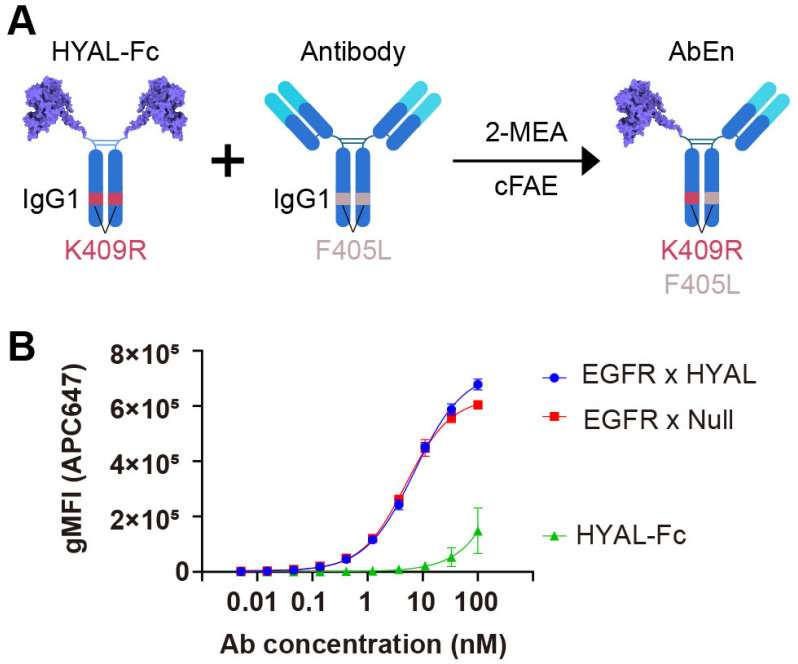
Engineering of an antibody–enzyme conjugate. (**A**) Schematic illustration of the generation of an antibody–enzyme conjugate (AbEn) via cFAE. The hyaluronidase-Fc fusion protein containing K409R mutation was incubated with an IgG1 molecule harboring matching F405L mutation in the presence of 2-MEA. (**B**) Concentration–response curves of the AbEn molecule binding to HCC827 cells were analyzed with Prism 8.0 using a four-parameter logistic equation. The data were presented as the mean ± SEM values of at least three independent experiments. The curve for EGFR × HYAL AbEn were shown in blue. The EGFR × Null antibody (red) and HYAL-Fc IgG1 fusion protein (green) served as the positive and negative controls, respectively. The abbreviations used: gMFI, geometrical mean fluorescent intensity; SEM, standard error of the mean; Fc, fragment crystallizable; AbEn, antibody–enzyme conjugate; cFAE, controlled Fab-arm exchange; 2-MEA, 2-mercaptoethylamine.

The AbEn was generated by fusing the human hyaluronidase PH20 domain (36–482) onto the hinge region of a human IgG1 Fc using a flexible GGGGS linker (Figure 1A, Supplementary Figure S1A). The resulting hyaluronidase-Fc (HYAL-Fc) construct had an average protein yield of 30 mg/L from the transient CHO expression system and demonstrated over 95% monomeric purity following a one-step protein A chromatography purification (Supplementary Figure S1B,C). In comparison to hyaluronidase with a C-terminal histidine tag (HYAL-His, 11,101 U/mg, 5,863 U/nM), the HYAL-Fc demonstrated comparable enzyme activity of 41,310 U/mg (6,352 U/nM) in a turbidity assay, confirming that the Fc region did not interfere with enzyme function (Supplementary Table S1).

Subsequently, we generated an AbEn (Supplementary Figure S2A,B) by combining an anti-EGFR antibody with HYAL-Fc using the controlled Fab-arm exchange (cFAE) technique [33]. The bispecific EGFR x HYAL AbEn was confirmed to be greater than 95% (Supplementary Figure S2A,B). To evaluate whether the AbEn retained its target-binding capability, we conducted a flow cytometry-based cell binding assay using the EGFR-expressing cancer cell line HCC827. The HYAL-Fc fusion protein alone demonstrated no binding to HCC827 cells, while the EGFR × HYAL bispecific agent exhibited comparable binding potency (reflected by EC_50_, 7.18 nM vs. 4.99 nM) and efficacy (reflected by E_max_, 7.26 × 10^5^ vs. 6.26 × 10^5^) to that of the EGFR × Null control bispecific antibody (Figure 1B, Supplementary Table S2). Thus, the enzyme domain fusion did not interfere with cancer target binding. Furthermore, the bispecific EGFR × HYAL AbEn maintained a single enzyme activity of 2,047 U/nM (13,670 U/mg), nearly corresponding to half of that of the parental HYAL-Fc protein containing two enzyme domains (Supplementary Table S1). Therefore, neither the cFAE process nor the anti-EGFR Fab domain adversely affected the enzymatic function. Collectively, these results confirmed the dual functionality of this novel AbEn, which retained both antigen-binding and enzyme activity properties.

### 3.2. Targeted Hyaluronidase Demonstrated Effective Depletion of HA Both Ex Vivo and In Vivo

Hyaluronidase can effectively degrade HA within the tumor stroma, resulting in a delay in tumor growth in nonclinical models across various solid tumor types [26,36,37]. Furthermore, its antitumor activity corresponded to the extent of HA present in the tumor [38]. To characterize the expression patterns of HA in the stroma of tumor, Alcian blue staining [39] was conducted on various cell line-derived xenograft (CDX) models. Among them, a subset of cells including the colorectal cancer cell line RKO, the lung cancer cell line HCC827, the pancreatic cancer cell line AsPC-1, and the gastric cancer cell line N87 exhibited HA-rich staining (Supplementary Table S3).

**Figure 2:**
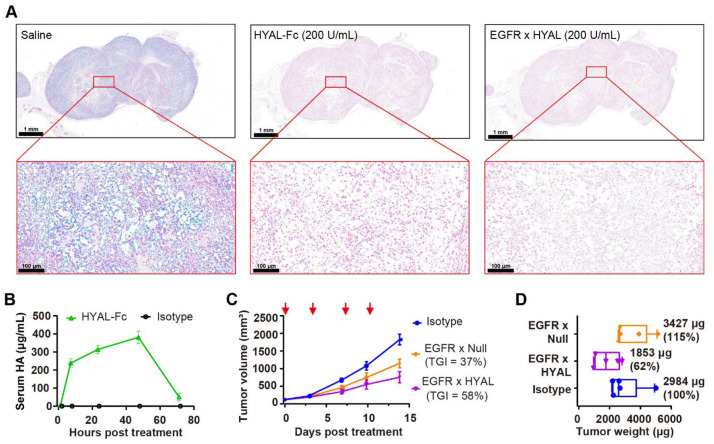
AbEn demonstrated effective depletion of HA both ex vivo and in vivo. (**A**) Alcian Blue staining of RKO tumor sections revealed changes in HA content following treatment with HYAL-Fc IgG1 fusion protein and EGFR × HYAL AbEn at a concentration of 200 U/mL for 2 h at 37 °C. The saline treatment served as a negative control. Alcian Blue specifically stained HA in the tissues (blue staining). The scale bars spanned for the top images, 1 mm; and for the bottom images, 100 μm. (**B**) Time course of serum HA fragment levels after a single intratumoral injection of 1 mg/kg HYAL-Fc IgG1 fusion protein in mice bearing subcutaneous RKO tumors. Blood samples (20 μL) were collected at 2, 8, 24, 48, and 72 h post-administration, and serum HA fragments were quantified via ELISA. Each time point included the mean values of *n* = 3 mice. An IgG1 isotype control was used as a negative control. (**C**) Tumor growth curves of RKO xenografts treated with either IgG1 isotype control (blue), EGFR × Null (orange), or EGFR × HYAL (purple) at 5 mg/kg, administered twice weekly for a total of four doses. The red arrows indicated the specific dosing days. Tumor growth inhibition rates were calculated relative to the isotype control for each treatment group for comparison. *n* = 5 mice per group. (**D**) On day 14, the animals were sacrificed, and the respective tumor weights were measured. Tumor weights were normalized to the isotype control for comparison. *n* = 5 tumors per group. The abbreviations used: HA, hyaluronic acid; TGI: tumor growth inhibition; HYAL, hyaluronidase; EGFR, epidermal growth factor receptor; AbEn, antibody–enzyme conjugate.

Next, the enzyme activity of the AbEn in both the ex vivo and in vivo HA-rich RKO models were determined. The alcian blue staining on RKO tumor Formalin-Fixed Paraffin-Embedded (FFPE) section slides, with or without the pre-treatment of HYAL-Fc or EGFR × HYAL before the staining, demonstrated that both HYAL-Fc and EGFR × HYAL effectively degraded HA in the RKO tumor stromata (Figure 2A). Additionally, the in vivo enzyme activity of HYAL-Fc, via monitoring the time course of HA degradation, was evaluated following the injection into RKO tumor-bearing mice. Serum HA fragments were detectable as early as 2 h after HYAL-Fc treatment, increased until 48 h, and then sharply declined at 72 h. This pattern of dynamic homeostasis of HA degradation and clearance in vivo was shown in Figure 2B. This prolonged HA degradation activity was also corroborated by PEGPH20-mediated HA removal, which persisted for over 72 h in a prostate cancer xenograft model [40]. Moreover, a pharmacokinetic (PK) study to monitor the half-life of the HYAL-Fc fusion protein demonstrated a serum half-life of 132 h, which was comparable to that of the human IgG1 isotype control (t_1/2_ = 157 h, Supplementary Figure S3). These results indicated that the enzyme domain did not affect FcRn binding and that the HYAL-Fc fusion protein was stable in circulation. In comparison to the pegylation strategy, which extended the serum half-life of PH20 from 3 min to approximately 10.3 h in mice [41], the Fc-fusion strategy produced a longer-acting hyaluronidase that could have advantages in facilitating sustained enzyme activity and reducing the frequency of administration.

To better distinguish the antitumor activity mediated by HA degradation from Fc effector functions, we introduced L234A and L235A (LALA) mutations into the CH2 domain of Fc to minimize the binding to Fc gamma receptors and C1q [42]. In HA-rich RKO tumors, intravenous administration of EGFR × HYAL effectively inhibited tumor growth, achieving a tumor growth inhibition (TGI) of 58% compared to 37% for EGFR × Null (Figure 2C), without affecting the mice’s body weight (Supplementary Figure S4). Notably, a 38% reduction in tumor weight was observed following treatment with EGFR × HYAL, whereas no notable change was noted with EGFR × Null, highlighting the specific role of hyaluronidase in remodeling the tumor stroma (Figure 2D). Together, the AbEn was capable of effectively degrading HA and delaying tumor growth in HA-rich tumors. There was no difference in tumor growth inhibition between the WT Fc and the LALA IgG1 Fc.

### 3.3. Enhancing Anti-Tumor Activities Through Targeted Hyaluronidase Delivery

Having established that the AbEn could mediate enzymatic activity in vivo, we next explored whether antibody-mediated delivery of hyaluronidase enhanced HA degradation efficiency and antitumor efficacy compared to systemic hyaluronidase treatment. Given the low-EGFR and high-PD-L1 expression profiles of the RKO cell line, we generated a novel PD-L1 × HYAL AbEn using the cFAE technology to improve tumor specificity (Supplementary Figure S2C,D). As expected, the PD-L1 × HYAL AbEn exhibited superior binding efficacy to RKO cells by having a higher E_max_ value of 1.00 × 10^6^ when compared to the EGFR × HYAL Aben with an E_max_ value of 1.42 × 10^4^ (Supplementary Figure S5).

**Figure 3:**
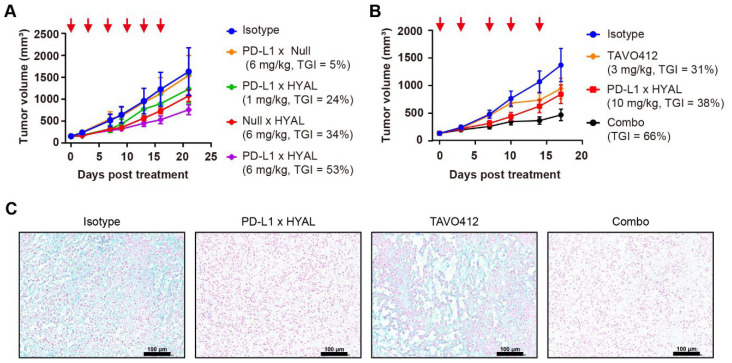
Targeted hyaluronidase enhanced the anti-tumor activities. (**A**) Tumor growth curves of RKO xenografts treated with IgG1 isotype control (blue), PD-L1 × Null (orange), or Null × HYAL (red) at 6 mg/kg, and PD-L1 × HYAL at 1 mg/kg (green) and 6 mg/kg (purple), administered twice weekly for six doses. Tumor growth inhibition rates were calculated relative to the isotype control for each treatment group. Each group included *n* = 5 mice. The red arrows indicated the specific dosing days. (**B**) Tumor growth curves of RKO xenografts treated with IgG1 isotype control (blue), TAVO412 (orange, 3 mg/kg for 4 doses and 5 mg/kg for fifth dose), PD-L1 × HYAL (red, 10 mg/kg), and the combination of PD-L1 × HYAL and TAVO412 (black), administered twice weekly for a total of five doses. The TGI rates were calculated relative to the isotype control for comparison. Each group included *n* = 5 mice. The red arrows indicated the specific dosing days. (**C**) Alcian Blue staining of RKO tumor sections demonstrated changes in hyaluronic acid (HA) content following treatment with IgG1 isotype control, PD-L1 × HYAL, TAVO412, the combination of PD-L1 × HYAL and TAVO412 (5 mg/kg). The scale bars spanned 100 μm. The abbreviations used: TGI, tumor growth inhibition; Combo, combination; HYAL, hyaluronidase.

Moreover, RKO tumors exhibited a dose-dependent response to PD-L1 × HYAL treatment, showing TGI levels of 24% at 1 mg/kg and 53% at 6 mg/kg dosing, respectively (Figure 3A). In contrast, systemic administration of hyaluronidase resulted in lower TGI values for the HYAL × Null (34%) and PD-L1 × Null control (5%); both being dosed at 6 mg/kg, respectively (Figure 3A). Thus, the targeted delivery of hyaluronidase through a tumor-associated antigen (TAA) mediated more effective HA degradation and enhanced antitumor responses than the responses coming from systemic administration.

Our studies revealed that the AbEn facilitated HA degradation in the tumor stroma, highlighting its potential role in enhancing the therapeutic efficacy of oncological agents. To explore this, we selected TAVO412, a novel EGFR × cMet × VEGF trispecific antibody known for its potent tumor growth inhibition across multiple solid tumors [43,44,45]. In RKO tumor-bearing mice, PD-L1 × HYAL plus TAVO412 produced a stronger antitumor response (TGI = 66%) compared to either treatment alone: TAVO412 (TGI = 31%) or PD-L1 × HYAL (TGI = 38%) (Figure 3B). Tumor tissues from treated mice were collected at the time of sacrifice for intratumoral HA quantification, and tissue sections were also stained with Alcian blue. PD-L1 × HYAL administered alone or with TAVO412, effectively removed HA in RKO xenograft tissue (Supplementary Figure S6A, Figure 3C). In contrast, HA depletion was not observed with TAVO412 treatment alone, indicating that the enhanced antitumor responses from the combination therapy were linked to HA degradation (Supplementary Figure S6A, Figure 3C). Collectively, these findings demonstrated that combining oncological therapeutics with targeted HA degradation strategies could significantly improve their efficacy.

### 3.4. Enhancing Anti-Tumor Activities Through CAF-Targeted Hyaluronidase Delivery

**Figure 4:**
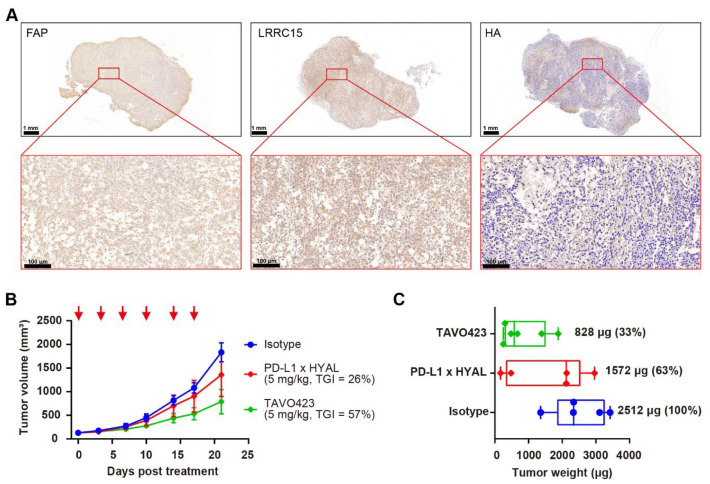
Targeted hyaluronidase delivery to stroma targets improved anti-tumor activity. (**A**) Representative immunohistochemistry images of RKO tumor sections showing FAP, LRRC15, and HA expression. The scale bar spans 100 μm. (**B**) Tumor growth curves of RKO xenografts treated with IgG1 isotype control (blue), PD-L1 × HYAL (red), or TAVO423 (green) at 5 mg/kg, administered twice weekly for six doses. Tumor growth inhibition rates were calculated relative to the isotype control. The data show an average of *n* = 5–6 mice per group. The red arrows indicate the specific dosing days. (**C**) On day 21, the animals were sacrificed and the respective tumor weights were measured. Tumor weights were normalized to the isotype control for comparison. Each group represents *n* = 5–6 mice per group. The abbreviations used: TGI, tumor growth inhibition; HYAL, hyaluronidase.

Given the enrichment of CAFs with the stroma in the TME, we hypothesized that delivering hyaluronidase via CAF targeting could achieve more effective HA degradation than targeting TAAs in solid tumor cells. Two CAF-specific targets, FAP and LRRC15, were selected to facilitate targeted delivery of hyaluronidase into the stroma. RKO tumor FFPE sections were stained with antibodies against FAP and LRRC15, along with HA-binding protein (HABP) to confirm the high levels of FAP and LRRC15 proteins in the tumor samples with a concomitant abundance of HA within the TME (Figure 4A).

To improve binding affinity to CAFs, we engineered a bispecific antibody by fusing tandem anti-LRRC15 VHH antibodies to the C-terminus of the heavy chain of an anti-FAP antibody. The anti-FAP antibody showed comparable binding affinity for human (EC_50_ = 0.08 nM) and mouse antigens (EC_50_ = 0.06 nM), while the anti-LRRC15 antibodies had slightly lower affinity for mouse LRRC15 (EC_50_ = 13.58 nM) compared to human LRRC15 (EC_50_ = 0.1326 nM); (Supplementary Figure S7, Supplementary Table S4). This cross-reactivity was crucial for evaluating targeted hyaluronidase delivery in mouse xenograft models, given that stromal components were of mouse origin. In flow cytometry assays using U-87 MG human glioma cells expressing both FAP and LRRC15, the dual-targeted antibody demonstrated increased binding (EC_50_ = 0.2645 nM vs. 12.68 nM and 0.8885 nM) compared to the FAP-only mAb (EC_50_ = 12.68 nM) and LRRC15-only mAb (EC_50_ = 0.8885 nM) controls (Supplementary Figure S7, Supplementary Table S4).

Subsequently, TAVO423, a trispecific FAP × LRRC15 × HYAL AbEn with an Fc-engineered IgG1 backbone carrying LALA mutations was generated using cFAE technology for in vivo proof of concept experiments (Supplementary Figure S8). In RKO tumor-bearing mice, TAVO423 demonstrated a superior antitumor effect (TGI = 57%) compared to PD-L1 × HYAL (TGI = 26%) when administered at the same dose (Figure 4B). Additionally, tumor tissues were collected at sacrifice to measure tumor weights, revealing a 67% reduction in tumor weight with TAVO423 treatment, compared to a 37% reduction with PD-L1 × HYAL treatment (Figure 4C). These results demonstrated a more pronounced tumor control effect, potentially driven by more extensive remodeling of the TME and alterations in tissue density and composition. Overall, the targeted hyaluronidase delivery via CAF antigens could disrupt the stromal barrier more effectively than strategies solely targeting TAAs, thereby enhancing antitumor responses.

### 3.5. CAF-Targeted HA Degradation Improves the Antitumor Responses of Diverse Antitumor Drugs in RKO Xenograft Model

Although being used as a standard of care drug for patients suffering from colorectal cancer (CRC), 5-fluorouracil (5-FU) chemotherapy has limited improvement in disease-free survival, largely due to TME-mediated chemoresistance and impaired drug penetration [46]. Similarly, while PD-1/PD-L1 inhibitors are approved for CRC, most patients experience poor responses or relapse due to TME-driven immunosuppression [47]. Recognizing HA accumulation as a common mediator of both chemoresistance and immunosuppression, we hypothesized that CAF-targeted HA degradation could potentiate existing therapies by inducing TME remodeling.

**Figure 5:**
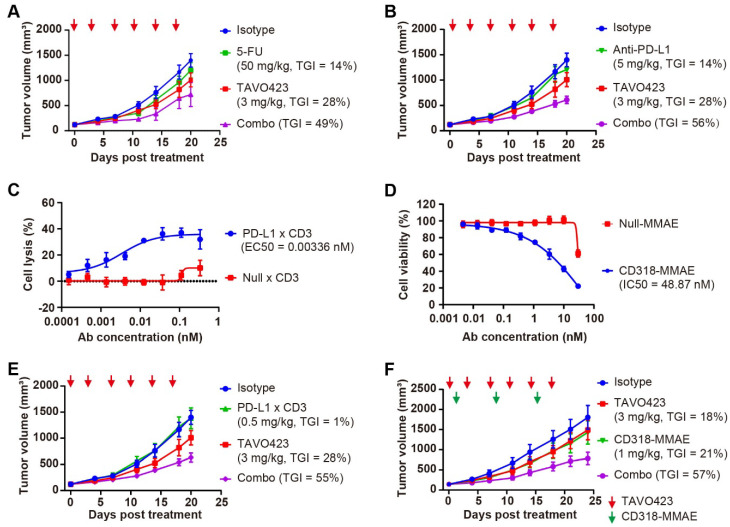
CAF-targeted HA degradation improved the antitumor responses of diverse anti-tumor drugs. (**A**) Tumor growth curves of RKO xenografts treated with IgG1 isotype control (3 mg/kg, blue), 5-FU (50 mg/kg, green), or TAVO423 (3 mg/kg, red), and a combination of 5-FU with TAVO423 (purple). Treatments were administered twice weekly for six doses. (**B**) Tumor growth curves of RKO xenografts in NOG mice reconstituted with human PBMCs, treated with IgG1 isotype control (3 mg/kg, blue), anti-PD-L1 monoclonal antibody (5 mg/kg, green), or TAVO423 (3 mg/kg, red), and a combination of anti-PD-L1 mAb and TAVO423 (purple). Treatments were administered twice weekly for six doses. (**C**) Representative concentration–response curve demonstrating PD-L1 × CD3-induced lysis of RKO cells (E:T ratio = 5:1). Cytotoxicity was measured via LDH release after 72 h of cocultures of PBMCs and RKO cells at an E:T ratio of 5:1, compared to the 0.2% (*w*/*v*) Triton X-100 control. The Null × CD3 antibody served as negative control. EC_50_ values were determined by four-parameter logistic curve fitting. Data are mean ± SEM values from at least three independent experiments. (**D**) Representative concentration–response curve showing CD318-MMAE-induced proliferation inhibition of RKO cells. Cytotoxicity was assessed for 4 days post-treatment. Null-MMAE served as a negative control. IC_50_ values were calculated using four-parameter logistic curve fitting. The data represented the mean ± SEM values from at least three independent experiments. (**E**) Tumor growth curves of RKO xenografts in NOG mice reconstituted with human PBMCs, treated with IgG1 isotype control (3 mg/kg, blue), PD-L1 × CD3 (0.5 mg/kg, green), or TAVO423 (3 mg/kg, red), and a combination of PD-L1 × CD3 and TAVO423 (purple). Treatments were administered twice weekly for six doses. (**F**) Tumor growth curves of RKO xenografts treated with IgG1 isotype control (3 mg/kg, blue), CD318–MMAE (1 mg/kg, green), or TAVO423 (3 mg/kg, red), along with a combination of CD318–MMAE and TAVO423 (purple). Treatment with TAVO423 was administered thrice weekly for 9 doses, and CD318-MMAE was given weekly for 3 doses. For all animal studies, tumor growth inhibition (TGI) rates were calculated relative to the isotype control. The studies showed the average of *n* = 5–6 mice per group. The red and green arrows indicated the specific dosing days. The abbreviations used: 5-FU, 5-Fluorouracil; PBMCs, peripheral blood mononuclear cells; mAb, monoclonal antibody; TGI, tumor growth inhibition; Combo, combination; EC_50_, median effective concentration; IC_50_, half-maximal inhibitory concentration; E:T, effector to target ratio; SEM, standard error of the mean.

The in vivo models using RKO tumor-bearing mice revealed that while TAVO423 and 5-FU monotherapies achieved modest TGI of 28% and 14%, respectively, their combination synergistically enhanced TGI to 49% (Figure 5A, Supplementary Table S5). The combination therapy also induced rapid and significant HA depletion in the tumor stroma within 0.25 to 1 h of treatment (Supplementary Figure S6B). A similar readout was observed in the experiment involving immune checkpoint blockade. In the HA-rich RKO tumors, the anti-PD-L1 monotherapy (TGI = 14%) was substantially improved in the combination with TAVO423 (TGI = 56%) (Figure 5B, Supplementary Table S5). These findings showed how targeted HA degradation could improve current CRC therapies by disruption of stromal barriers.

Since HA degradation has been shown to enhance drug penetration and immune infiltration [26,48], we expanded our investigation to include T-cell engagers (TCEs) and antibody–drug conjugates (ADCs). Both modalities could benefit from having stronger activity through more efficient tumor penetration and immune recruitment. Leveraging the expression profiles of the RKO cell line, we engineered a bispecific TCE targeting PD-L1 and CD3, as well as a novel ADC targeting CD318. CD318, also known as cub domain containing protein 1 (CDCP1), was overexpressed in many solid tumors and could be a promising target in treating colorectal tumors [49]. The PD-L1 × CD3 bispecific antibody comprised an anti-PD-L1 VHH and an anti-CD3 IgG1 domains. The CD318-MMAE ADC was created by conjugating an anti-CD318 IgG1 to the anti-mitotic toxin payload monomethyl auristatin E (MMAE), via a lysosomal cleavable dipeptide linker. Both constructs incorporated Fc-engineered IgG1 (LALA mutants) to reduce Fcγ receptor (FcγR) interactions.

To assess target engagement and efficacy prior to in vivo testing, the anti-tumor effects were assessed using two different in vitro cytotoxicity assay formats. The PD-L1 × CD3 TCE exhibited potent tumor killing (EC_50_ = 0.00336 nM) in RKO-PBMC cocultures (Figure 5C), while the CD318-MMAE ADC showed nanomolar growth inhibition (IC_50_ = 48.87 nM) against RKO monolayers (Figure 5D). In subsequent in vivo studies using PBMC-humanized RKO xenografts, administering PD-L1 × CD3 (0.5 mg/kg) or TAVO423 (3 mg/kg) alone twice weekly for six doses, showed limited activity with TGI values of 1% and 28%, respectively. However, the combination of TAVO423 and PD-L1 x CD3 achieved a TGI of 55% (Figure 5E, Supplementary Table S5). Similarly, in RKO xenografts, combining targeted HA degradation (3 mg/kg, administered thrice weekly for nine doses) with the CD318-MMAE ADC (1 mg/kg, weekly for three doses) resulted in superior efficacy, reaching 67% TGI versus 18–21% with either agent alone (Figure 5F, Supplementary Table S5). Together, these findings further supported how CAF-targeted HA degradation could remodel the TME to synergize TGI with various therapeutic modalities in HA-rich CRC tumors.

To further validate the broad applicability of CAF-targeted HA degradation across diverse tumor types, we assessed TAVO423 in a pancreatic cancer model employing BxPC-3 cells engineered to overexpress hyaluronan synthase 3 (BxPC-3-HAS3). These cells exhibited a 17-fold increase in intratumoral HA accumulation compared to wild-type (WT) BxPC-3 controls (Supplementary Figure S9A,B). In this HA-high setting, TAVO423 monotherapy (5 mg/kg) achieved moderate tumor growth inhibition (TGI = 30%) and reduced intratumoral HA levels by 5-fold (Supplementary Figure S9B,C). While a suboptimal dose of 5T4 × CD3 TCE (0.1 or 0.5 mg/kg) alone showed substantial efficacy (TGI = 73%; Supplementary Figure S9C), the combination of TAVO423 with 5T4 × CD3 TCE led to superior antitumor activity (TGI = 92%), indicating that targeted HA degradation potently enhanced T-cell-mediated cytotoxicity in pancreatic tumors. All treatments were well tolerated in the rodent models as confirmed by minimal changes in the body weight throughout the study (Supplementary Figure S9D). Together, these results demonstrated the broad potential of CAF-directed HA degradation to augment diverse therapeutic modalities across multiple HA rich solid tumor types.

### 3.6. Targeted Hyaluronic Acid Degradation Sensitizes Tumors to Immune Checkpoint Blockade Therapy by Promoting the T Cells Infiltration

**Figure 6:**
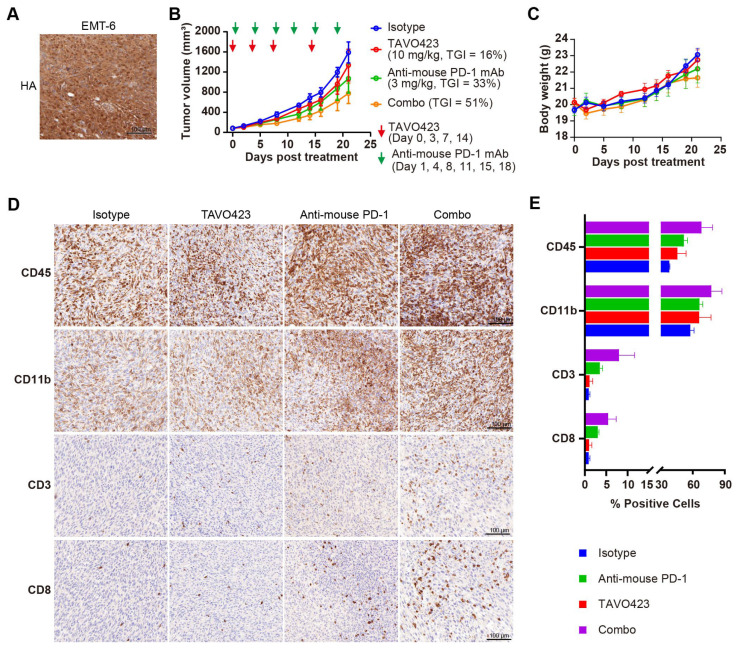
TAVO423 enhanced immune checkpoint blockade therapy by promoting T cell infiltration. (**A**) Representative immunohistochemistry images of EMT-6 tumor sections stained with HA-binding protein, demonstrating HA expression. Scale bars: 100 μm. (**B**,**C**) Balb/c mice were subcutaneously (s.c.) inoculated with 5 × 10^5^ EMT-6 tumor cells and treated as follows: 10 mg/kg TAVO423 on days 0, 3, 7, and 14; 3 mg/kg anti-mouse PD-1 mAb twice weekly on days 1, 4, 8, 11, 15, and 18; and a combination of TAVO423 plus anti-mouse PD-1 mAb with the same dosage and regimen as monotherapy. The red and green arrows indicated the specific dosing days. Tumor growth curves (**B**) and mouse body weights (**C**) of EMT-6-bearing mice were shown for groups receiving the IgG1 isotype control (blue), TAVO423 (red), anti-mouse PD-1 mAb (green), and combination therapy (orange). Tumor growth inhibition rates were calculated relative to the isotype control. The studies were an average of *n* = 5 mice per group. (**D**,**E**) At day 21 post-treatment, mice were sacrificed, and tumor tissues were collected for immunohistochemical analysis of intratumoral levels of CD3, CD8, CD45, and CD11b. (**D**) Representative IHC images of EMT-6 tumor sections. Scale bar: 100 μm. (**E**) Quantitative analysis of the percentage of CD3, CD8, CD45, and CD11b positive cells in tumor samples. The X-axis showed a broken scale with a gap between 15% and 30% to accommodate the data range, while maintaining a consistent linear scale throughout. All bars were color-coded as in the legend (Isotype: blue; Anti-mouse PD-1: green; TAVO423: red; Combo: purple). Results from two independent experiments are shown as mean ± SEM values. The abbreviations used: mAb, monoclonal antibody; TGI, tumor growth inhibition; Combo, combination; IHC, immunohistochemistry; SEM, standard error of the mean. HA, hyaluronan acid.

Compelling clinical evidence demonstrated that sufficient amounts of tumor-infiltrating lymphocytes (TILs) were essential for PD-1/PD-L1 blockade response and improved prognoses in many solid tumors [50]. Targeted HA degradation enhancement of ICIs efficacy by facilitating TIL recruitment was assessed by characterizing TAVO423 trispecific AbEn in combination with anti-PD-1 therapy in immunocompetent mice bearing EMT-6 syngeneic breast tumors. Unlike human tumor xenografts requiring immunocompromised hosts, these syngeneic models maintained fully functional immune systems so as to assess immunotherapy mechanisms and efficacy [51]. In addition, the constructs were fully cross-reactive with mouse target antigen counterparts, allowing for more relevant evaluation in these syngeneic models. IHC staining revealed substantial HA accumulation within the EMT-6 TME (Figure 6A), confirming the relevance of the model for hyaluronidase-based intervention. Treatment with anti-PD-1 monotherapy (3 mg/kg) demonstrated the expected antitumor activity (33% TGI; Figure 6B) in this immunotherapy-responsive model [52]. While TAVO423 alone (10 mg/kg) achieved modest efficacy (TGI = 16%), the combination regimen exhibited synergistic tumor control (TGI = 51%) while maintaining an excellent safety profile as demonstrated by stable body weight measured during the study (Figure 6C). These findings demonstrated both the preliminary therapeutic potential and favorable tolerability of this CAF-targeted hyaluronidase approach in immunocompetent hosts.

To determine if the enhanced antitumor activity of the combination of anti-PD-1 antibody and HA degradation mediated increased levels of TILs, we collected and analyzed tumor tissues post-treatment using IHC staining with various immune cell markers, including CD45 for leukocytes, CD11b for myeloid cells, and CD3 and CD8 for T cells (Figure 6D). While treatment regimens did not significantly alter overall leukocyte or myeloid cell infiltration, there were profound differences in the T cell populations (Figure 6D,E, Supplementary Table S6). Compared to the isotype control, the anti-PD-1 monotherapy induced a 3–4-fold expansion of both CD3^+^ and CD8^+^ T cells. The combination therapy of targeted HA degradation with anti-PD-1 had a 6–9-fold increase in T cell infiltration (Figure 6D,E; Supplementary Table S6). This robust infiltration of CD8^+^ cytotoxic T lymphocytes (CTLs) could drive the improved tumor growth inhibition control. Notably, the TAVO423 monotherapy alone failed to increase TILs, consistent with published evidence that HA degradation strategy required the combination with T cell priming agents for optimal efficacy [53]. Thus, the combination of TAVO423 for targeted HA degradation and ICIs could result in remodeling of the TME to facilitate CTL infiltration thereby enhancing tumor growth inhibition.

## 4. Discussion

Tumor-targeting cell therapy often has limited efficacy because of the immunosuppressive and physically obstructive TME. Thus, there have been efforts to remodel the TME to enhance anti-cancer responses from immunotherapies [54,55]. The HA-rich desmoplastic stroma in the solid tumors are barriers that impede drug penetration and immune cell infiltration. Although systemic hyaluronidase therapy has demonstrated promising preclinical success [30], we hypothesized that an antibody-mediated tumor-targeted delivery strategy could enhance HA degradation while mitigating off-tumor toxicity from systemic exposure. To this end, we developed a HYAL-Fc fusion protein to extend the hyaluronidase serum half-life and provide tissue targeting using a multifunctional AbEn using the cFAE technology. The AbEn molecules were engineered to preserve both target-binding specificity and enzymatic activity. In HA-rich colorectal cancer models, the AbEn molecules demonstrated superior therapeutic efficacy compared to conventional systemic delivery of hyaluronidase through more efficient HA degradation. Notably, the CAF-targeted AbEns exhibited greater therapeutic potency than tumor cell-targeted AbEns and synergistically improved outcomes via combination with other multiple treatment modalities. Furthermore, tumor-infiltrating lymphocyte augmentation was confirmed as a key driver of the enhanced anti-PD-1 response. This targeted HA degradation could overcome ECM-mediated inhibition of diverse therapies to treat patients suffering from growth of stroma-rich solid tumors.

The AbEn platform could overcome the lack of clinical responses from earlier attempts at HA-targeted therapies. Preclinical studies of PEGPH20 demonstrate effective HA degradation and TME remodeling effects by improving vascular patency thereby allowing enhanced chemotherapeutic delivery [26]. However, the clinical translation fail to meet the primary endpoint and suffered from dose-limiting toxicities [30]. These setbacks highlight two key challenges: (1) the narrow therapeutic window of systemic hyaluronidase therapy, and (2) the inability of HA depletion alone to sustain antitumor responses. Furthermore, PEGPH20 also show that disrupting the stromal barrier lead to HA fragmentation that can exacerbate tumor inflammation [30,56].

To circumvent such challenges, we developed the targeted HA AbEn to have two key features: (1) Fc fusion and heterodimerization for precise IgG1-scaffold assembly, (2) manipulation of tumor-targeting domain and the tunable valency to balance tumor penetration and enzyme delivery. As a result, compared to the untargeted PEGPH20 monotherapy approach, the AbEn platform offered four transformative advantages: (1) targeted HA degradation to enhance antitumor responses while minimizing systemic toxicity; (2) CAF-directed delivery to allow better immune cell infiltration; (3) the IgG1 scaffold enabled an extended half-life and concomitant immune effector functions (e.g., Depletion of CAFs mediated by Fc effector function); and (4) modular design permitted ‘plug-and-play’ integration of additional mechanisms of action, including antiangiogenic or immune-checkpoint inhibition.

Recent advances to localize HA degradation include mesothelin-specific CAR-T cells expressing PH20 and oncolytic adenoviruses that encode PH20 (e.g., VCN-01) to improve T-cell infiltration and stromal modulation in preclinical models [37,48]. However, these approaches may have two critical limitations. First, their therapeutic activities require successful tumor homing and proliferation of CAR-T cells or viral replication that can be compromised in densely fibrotic tumors. Second, continuous PH20 production by these living systems may lead to systemic enzyme dissemination which can raise safety concerns from off-tumor tissue damage. In contrast, our AbEn strategy did not depend on biological amplification, but rather allowed dose control for precise spatiotemporal control of HA degradation. This ‘on-demand’ mechanism offered distinct advantages including reduced systemic toxicity, streamlined manufacturing, and predictable pharmacokinetics to address key challenges in clinical translation.

Despite these promising findings, our study had several limitations that warrant consideration. First, although CAF-directed HA degradation demonstrated enhanced anti-tumor efficacy, we could not determine whether this resulted from CAF apoptosis or functional TME reprogramming. This distinction was critical because complete CAF elimination could unexpectedly accelerate tumor progression in certain contexts [57,58]. Future studies employing single-cell RNA sequencing and functional assays (e.g., contractility measurements) could elucidate these mechanistic details. Second, while co-expression of FAP and LRRC15 was common on pro-fibrotic CAFs, neither marker captured the full complexity of CAFs [17,59]. Third, although our PK data indicated prolonged serum stability of HYAL-Fc and efficacy studies in immunocompetent models demonstrated acceptable safety profiles, a comprehensive evaluation in non-human primates would be essential to assess potential off-tumor effects in HA-rich tissues (e.g., synovium, skin) and FAP (e.g., bone marrow) or LRRC15 (e.g., hair follicles) expressing sites. Finally, in the analysis of immune cell infiltration, the increase in CD8^+^ T cells could not be enough for robust anti-tumor activity in clinical settings. We did not characterize other T cell subsets, such as regulatory T cells (Tregs), within the total CD3^+^ population, nor did we dissect the heterogeneous CD11b^+^ myeloid compartment into its pro- and anti-inflammatory subsets. A more detailed immune profiling in future studies could provide deeper insights into the immunomodulatory mechanisms of targeted HA degradation. Additionally, while our syngeneic models effectively demonstrated increased immune infiltration, they could not fully capture human stromal heterogeneity. More clinically relevant systems, such as patient-derived organoid/CAF cocultures, could improve translational predictability. Addressing these gaps would be critical for both fundamental understanding of biology and subsequent clinical development.

## 5. Conclusions

In conclusion, our study demonstrated that targeted HA degradation can overcome ECM-mediated treatment resistance to enhance tumor growth inhibition. The AbEn platform’s modularity not only addressed key limitations of existing hyaluronidase base tumor growth inhibition approaches (e.g., short half-life, narrow therapeutic window) but also provided a modular platform for precision ECM remodeling to optimize combination regimens against stroma-rich malignancies. Notably, the PH20-based selective enzymatic activity for efficient HA degradation while minimizing off-target effects could enhance both safety and therapeutic efficacy and broaden the therapeutic index. Future studies exploring its application across diverse tumor types and stromal microenvironments could facilitate clinical implementation of this therapeutic paradigm. Further optimization of PH20 binding and catalytic efficiency could refine stromal targeting for both oncology, autoimmune, and inflammatory indications.

## Data Availability

Data will be made available upon reasonable request from the corresponding author.

## Supplementary Materials

The following supporting information can be downloaded at: https://www.mdpi.com/article/10.3390/cancers17213411/s1, Figure S1: Design and confirmation of the purified hyaluronidase-Fc fusion protein; Supplementary Figure S2: Generation of the TAA-targeted hyaluronidase AbEn molecules; Supplementary Figure S3: PK study of a hyaluronidase-Fc fusion protein in female BALB/c nude mice; Supplementary Figure S4: Effect of dosing on mouse body weights; Supplementary Figure S5: Comparison of PD-L1 binding with EGFR binding on the RKO cell line; Supplementary Figure S6: Quantification of intratumoral HA in RKO xenografts following treatment; Supplementary Figure S7: Binding of anti-FAP and anti-LRRC15 antibodies; Supplementary Figure S8: Engineering of a FAP x LRRC15 x HYAL AbEn, CAF-targeted hyaluronidase; Supplementary Figure S9: TAVO423 effects on the TGI efficacy of a 5T4 × CD3 TCE in a hyaluronan-rich pancreatic cancer model; Supplementary Table S1: Hyaluronidase enzyme activity of HYAL-Fc, HYAL-His, and EGFR × HYAL AbEn; Supplementary Table S2: Binding of EGFR x HYAL AbEn to the HCC827 cell line; Supplementary Table S3: Target Profile Screening of CDX models with HA-high stroma; Supplementary Table S4: Binding properties of anti-FAP x anti-LRRC15 bispecific antibodies; Supplementary Table S5: Tumor growth inhibition values of TAVO423 combined with various antitumor drugs; Supplementary Table S6: Statistical analyses of immune cells populations of tumor samples via IHC staining; Supplementary Table S7: Profiles of the drugs and antibody constructs.

## Abbreviations

The following abbreviations are used in this manuscript:

Abbreviation	Definition
2-MEA	2-mercaptoethylamine
5-FU	5-Fluorouracil
ADCs	Antibody–drug conjugates
AbEn	Antibody–enzyme conjugate
ATCC	American Type Culture Collection
CAF	Cancer-associated fibroblast
CAR-T	Chimeric antigen receptor T
CD44	Cluster of differentiation 44
CDCP1	Cub domain containing protein 1
CDX	Cell line-derived xenograft
cFAE	Controlled Fab-arm exchange
CH2	Constant Heavy domain 2
CH3	Constant Heavy domain 3
CI	Confidence interval
Combo	Combination
CRC	Colorectal cancer
CTLs	Cytotoxic T lymphocytes
DMEM	Dulbecco’s modified Eagle’s medium
E:T	Effector-to-target
EC50	Median effective concentration
ECM	Extracellular matrix
EGFR	Epidermal growth factor receptor
ELISA	Enzyme linked immunosorbent assay
EMEM	Eagle’s Minimum Essential Medium
FAP	Fibroblast activation protein
FBS	Fetal bovine serum
Fc	Fragment crystallizable
FcγR	Fcγ receptor
FFPE	Formalin-fixed, paraffin-embedded
FITC	Fluorescein isothiocyanate
GGGGS	Glycine–Glycine–Glycine–Glycine–Serine
gMFI	Geometrical mean fluorescent intensity
HA	Hyaluronan or Hyaluronic acid
HABP	HA-binding protein
HAS3	Hyaluronan synthase 3
HC	Heavy chain
His	Histidine
HMW	High molecular weight
HPLC-SEC	High-performance liquid chromatography–size exclusion chromatography
HRP	Horseradish peroxidase
HYAL	Hyaluronidase
IACUC	Institutional Animal Care and Use Committee
IC50	Half-maximal inhibitory concentration
ICIs	Immune checkpoint inhibitors
IHC	Immunohistochemistry
LALA	L234A and L235A
LC	Light chain
LDH	Lactate dehydrogenase
LMW	Low molecular weight
LRRC15	Leucine-rich repeat-containing protein 15
mAbs	Monoclonal antibodies
mAU	Milliabsorbance
min	Minutes
MMAE	Monomethyl auristatin E
MW	Molecular weight
nM	Nanomolar
OD450	Optical density at 450 nm
PBMCs	Peripheral blood mononuclear cells
PEGPH20	Pegylated recombinant human PH20
PH20	Plasma membrane-associated Hyaluronidase 20, Sperm adhesion molecule 1
PK	Pharmacokinetics
RHAMM	Hyaluronan-mediated motility receptor
SDS-PAGE	Sodium dodecyl sulfate polyacrylamide gel electrophoresis
SEM	Standard error of the mean
TAA	Tumor-associated antigen
TCEs	T-cell engagers
TGI	Tumor growth inhibition
TILs	Tumor-infiltrating lymphocytes
TME	Tumor microenvironment
U	Units
UV280	Ultraviolet absorption at 280 nm
WT	Wild-type
