# Supplementary material for: Targeted Hyaluronan Degradation Enhanced Tumor Growth Inhibition in Gastrointestinal Cancer Models
Source: Cancers (Basel). 2025 Oct 23;17(21):3411. doi: 10.3390/cancers17213411 (PMC12610240; doi:10.3390/cancers17213411)
Supplement: Supplementary file 1 [file cancers-17-03411-s001.zip › cancers-3864348-supplementary.pdf]

Targeted Hyaluronan Degradation Enhanced Tumor Growth Inhibition in Gastrointestinal Cancer Models

Fulai Zhou [1], Guangmao Mu [1], Honglei Bi [1], Limin Chen [1], Zhengxia Zha [1], Ying Jin [1] and Mark L. Chiu [1,2,*]

[1] Research & Development Department, Tavotek Biotherapeutics, Suzhou 215000, China
[2] Research & Development, Tavotek Biotherapeutics, Spring House, PA 19102, USA
* Correspondence: mark.chiu@tavotek.com

Supplementary Materials—Figures and Tables

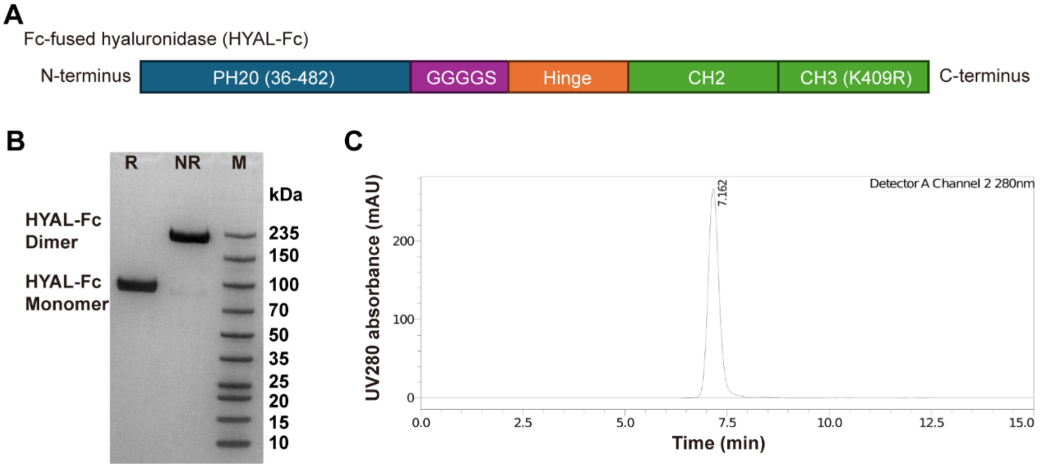

Supplementary Figure S1. Design and confirmation of the purified hyaluronidase-Fc fusion protein. (**A**) Schematic diagram of the structure of Fc-fused hyaluronidase (HYAL-Fc) expression construct. (**B**) Representative SDS-PAGE image of HYAL-Fc after Coomassie blue staining. (**C**) Representative size exclusion chromatography (SEC) profile of HYAL-Fc. **Abbreviations used:** M, SDS-PAGE molecular weight marker; NR, non-reducing; R, reducing; HYAL, hyaluronidase; Fc, fragment crystallizable region; mAU, milli-absorbance units; UV280, ultraviolet absorption at 280 nm; PH20, Plasma membrane-associated Hyaluronidase 20, also called sperm adhesion molecule 1; GGGGS, Glycine-Glycine-Glycine-Glycine-Serine; CH2, Constant Heavy domain 2; CH3, Constant Heavy domain 3; min, minutes.

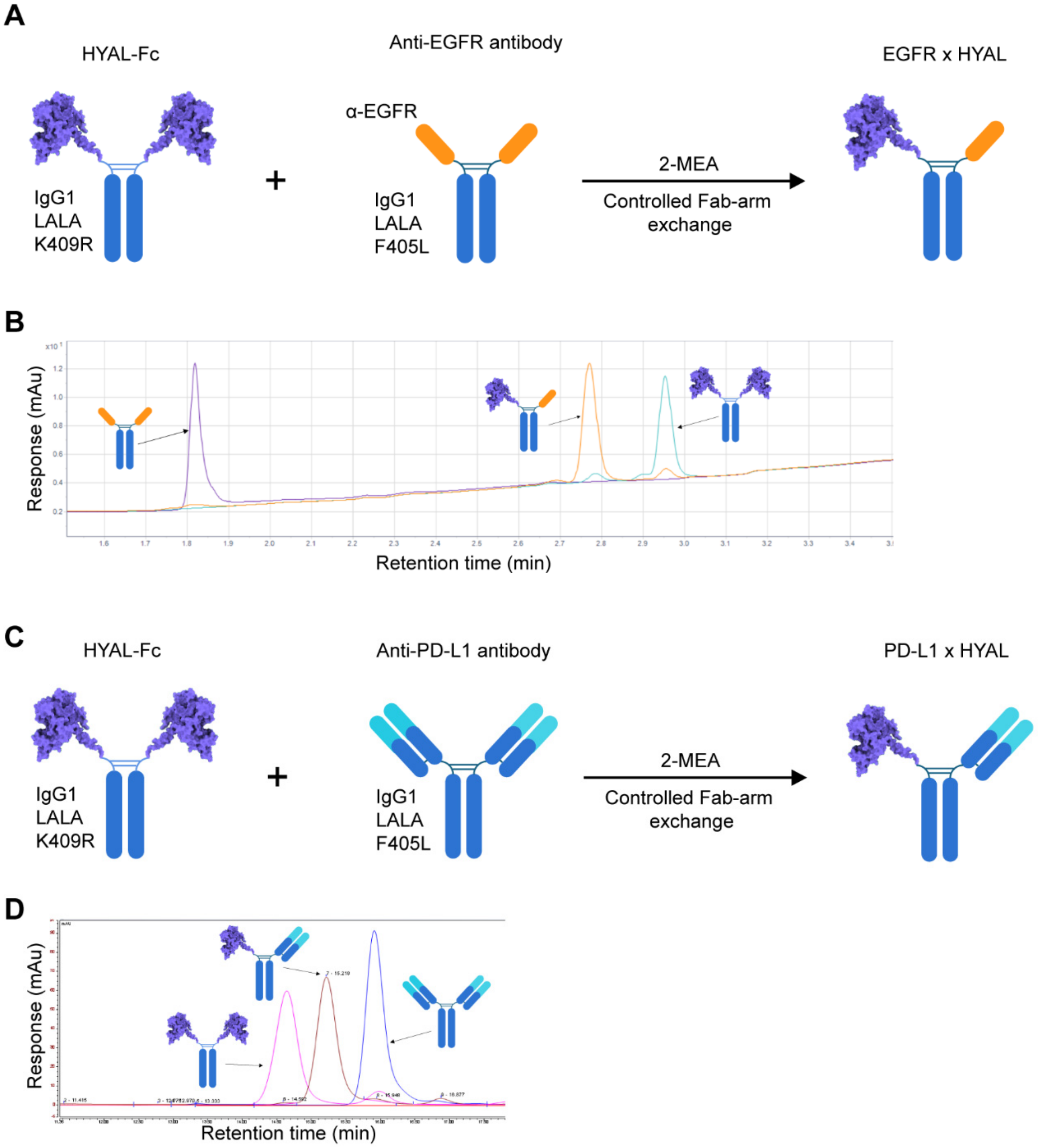

Supplementary Figure S2. Generation of the TAA-targeted hyaluronidase AbEn molecules. (**A-D**) Schematic illustration of *in vitro* generation of an EGFR x HYAL AbEn (A-B) or an PD-L1 x HYAL AbEn (C-D) via cFAE. Hyaluronidase-Fc (HYAL-Fc) fusion proteins with K409R mutation are incubated with an anti-EGFR or anti-PD-L1 antibody containing matching F405L mutation, in the presence of 2-MEA. HPLC-SEC analysis confirmed the bispecific AbEn formation, based on the molecular weight differences between the parental antibody or HYAL-Fc fusion and the resulting bispecific antibody–enzyme conjugate. The bispecific AbEn molecule exhibited an average molecular weight corresponding to the average molecular weight of the two parental molecules. The monomeric purity was assessed via HPLC-SEC on an Agilent 1260 Infinity II LC system using an AdvanceBio SEC column, with an isocratic method of 150 mM KH_2PO_4 buffer at pH 7.3, room temperature, and detection at 280 nm, where the monomeric peak area was calculated as a percentage of the total injection to confirm over 90% exchange efficiency. The corresponding peaks for the parental and bispecific AbEn are indicated in C and D. **Abbreviations used**: LALA referred to L234A and L235A mutations; HYAL, hyaluronidase; Fc, fragment crystallizable region; TAA, tumor-associated antigen; AbEn, antibody-enzyme fusion; cFAE, controlled Fab-arm exchange; 2-MEA, 2-mercaptoethylamine.

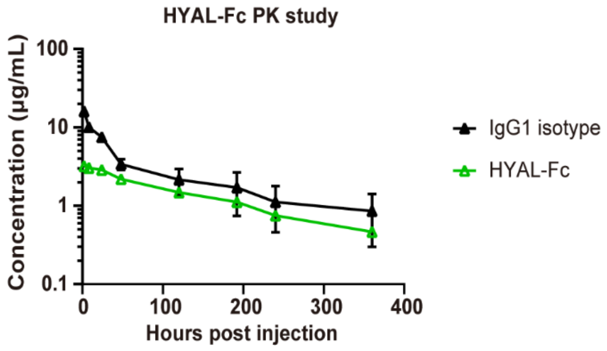

	Unit	IgG1 isotype	HYAL-Fc
$t_{1/2}$	Hours	157	132

Supplementary Figure S3. PK study of the hyaluronidase-Fc fusion protein in female BALB/c nude mice. Top graph: Mean plasma concentration over time for IgG1 isotype control and HYAL-Fc IgG1 fusion protein after 1 mg/kg intravenous (i.v.) administration. Bottom table beneath the graph displayed the calculated half-life ($t_1/_2$), indicating the time required for the drug concentration in the body to decrease by half during elimination. **Abbreviations used**: mg/mL, microgram per milliliter; $t_{1/2}$, half-life; HYAL, hyaluronidase; Fc, fragment crystallizable region; PK, pharmacokinetics.

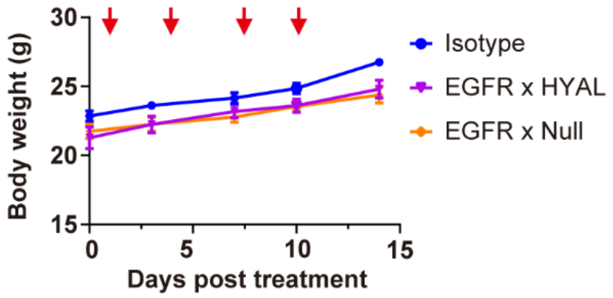

Supplementary Figure S4. Effect of dosing on mouse body weights. RKO xenografts treated with either IgG1 isotype control (blue), EGFR × Null (orange), or EGFR × HYAL (purple) at 5 mg/kg, administered twice weekly for four doses**.** n=5 mice per group. Abbreviations used: HYAL, hyaluronidase; EGFR, epidermal growth factor receptor.

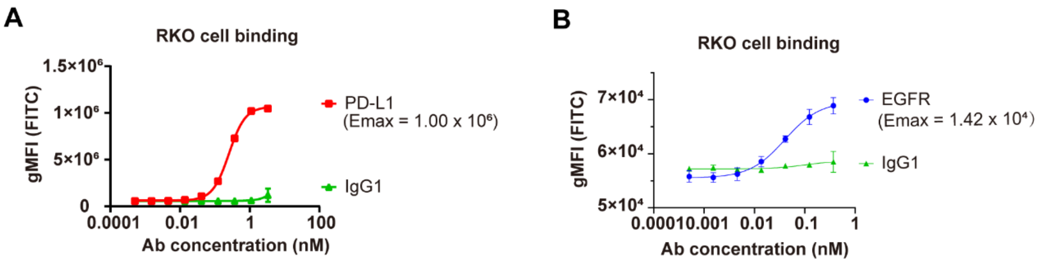

Supplementary Figure S5. Comparison of PD-L1 and EGFR binding on the RKO cell line.
A. RKO cell binding curves for the anti-PD-L1 mAb (red) and the IgG1 isotype control (green) as the negative control. **B.** RKO cell binding curves for the anti-EGFR mAb (blue) and the IgG1 isotype control (green) as the negative control. The cell binding was analyzed using a Prism 8.0 four-parameter logistic model to determine E_{max}. Data are shown as the mean ± SEM values from at least three independent experiments. **Abbreviations used**: gMFI: geometrical mean fluorescent intensity; SEM: standard error of the mean; E_{max}: maximal binding capacity; FITC, fluorescein isothiocyanate; nM, nanomolar; Ab, antibody.

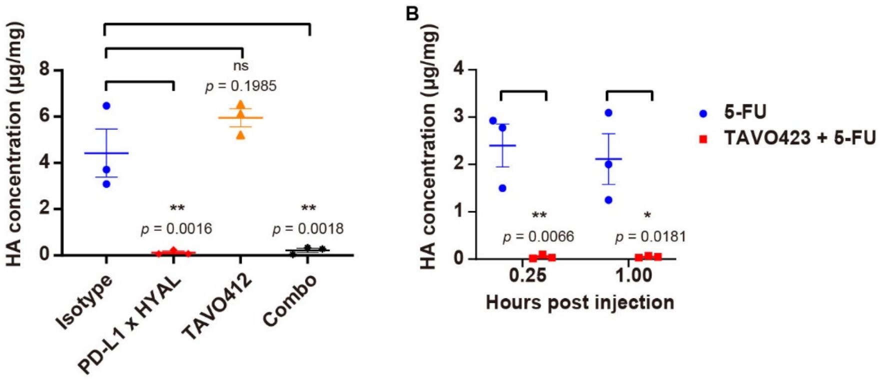

Supplementary Figure S6. Quantification of intratumoral HA in RKO xenografts following treatment. (**A**) Mice bearing RKO tumors were treated twice weekly with an IgG1 isotype control (blue), TAVO412 (3 mg/kg for four doses, then 5 mg/kg for the fifth dose; orange), or a PD-L1 × HYAL bispecific antibody (10 mg/kg; red) for a total of five doses. (B) Tumors were treated with 5-FU control (blue) or a combination of TAVO423 and 5-FU for 0.25 or 1 hour. (A and B) Tumors were collected at the endpoint or indicated times, and intratumoral HA was quantified by ELISA (n=3 per group) and normalized to the initial tumor weight, expressed as µg of HA per mg of tissue. Data are presented as the mean ± SEM values from at least three independent experiments. Significance was determined by one-way ANOVA (A) or an unpaired two-tailed t-test (B); *$p < 0.05$, **$p < 0.01$, ***$p < 0.001$. **Abbreviations used**: HA, hyaluronan; ns: not significant; Combo, combination; SEM: standard error of the mean; 5-FU, 5-fluorouracil; ELISA, enzyme linked immunosorbent assay; µg, microgram; mg, milligram; kg, kilogram

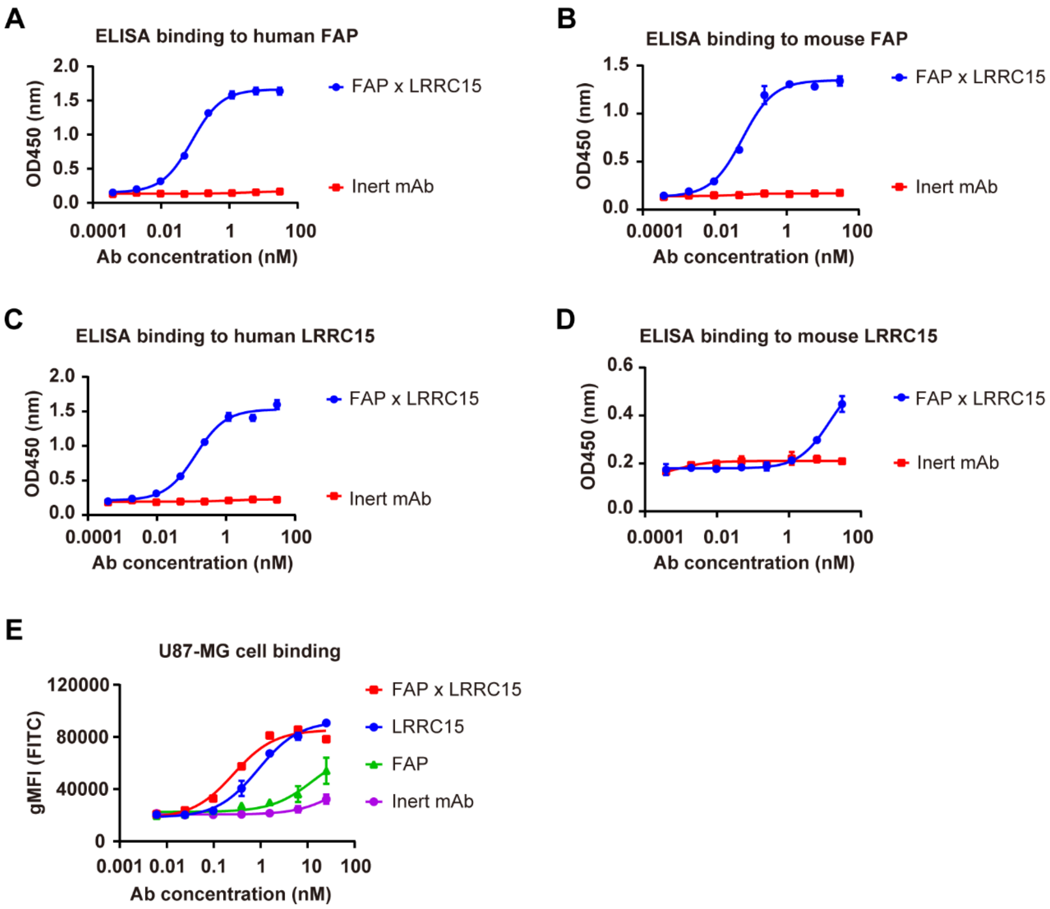

Supplementary Figure S7. Binding of the anti-FAP and anti-LRRC15 antibodies. ELISA binding curves of FAP x LRRC15 bispecific antibody (blue) and inert mAb (red) to (**A**) human FAP; (**B**) mouse FAP mAb, (**C**) human LRRC15, and (**D**) mouse LRRC15. The binding curves were analyzed using a Prism 8.0 four-parameter logistic model. (**E**) UG-MG cell binding curves for FAP x LRRC15 bispecific antibodies (red), anti-LRRC15 antibody (blue), anti-FAP antibody (green), and inert mAb (purple) analyzed with the same model. Data are presented as the mean ± SEM values from at least three independent experiments. **Abbreviations used**: gMFI: geometrical mean fluorescent intensity; SEM: standard error of the mean; mAb, monoclonal antibody; nM, nanomolar; OD450, optical density at 450 nm; ELISA, enzyme linked immunosorbent assay; FITC, fluorescein isothiocyanate.

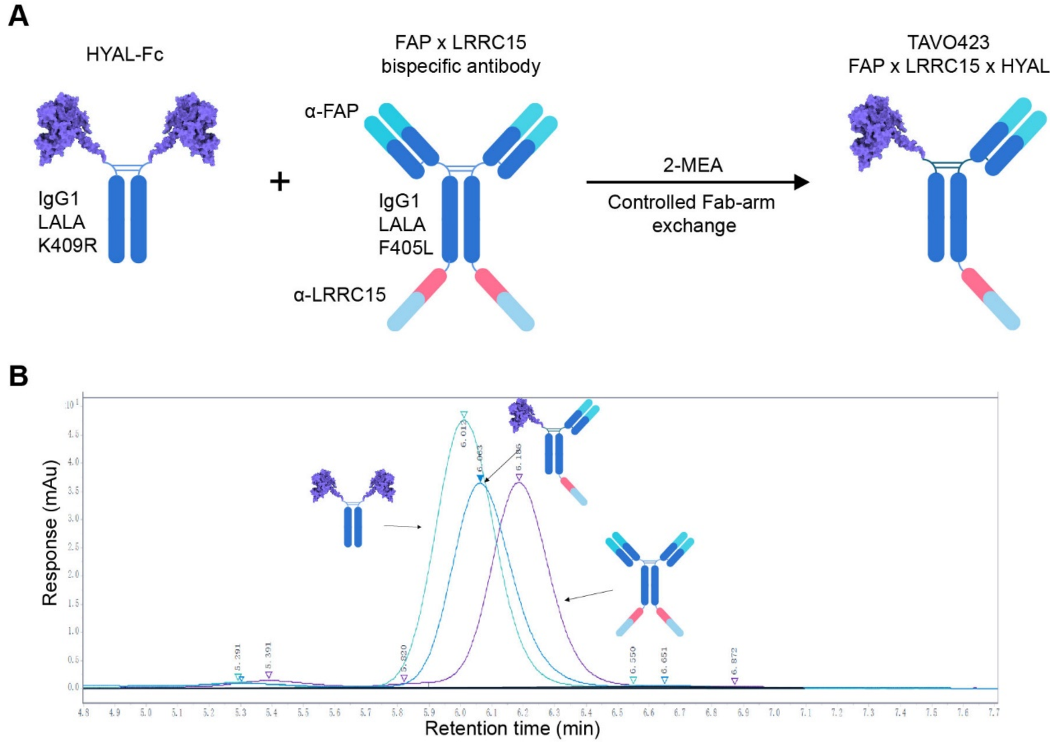

Supplementary Figure S8. Engineering of FAP x LRRC15 x HYAL AbEn, the CAF-targeted hyaluronidase. (**A**) Schematic illustration of the *in vitro* generation of an FAP x LRRC15 x HYAL AbEn (TAVO423) via cFAE. Hyaluronidase-Fc (HYAL-Fc) fusion proteins with K409R mutation are incubated with FAP x LRRC15 bispecific antibodies containing matching F405L mutation, in the presence of 2-MEA. (**B**) HPLC-SEC analysis confirmed the trispecific AbEn formation, based on the molecular weight differences between the parental antibody or HYAL-Fc fusion and the resulting bispecific antibody–enzyme conjugate. The trispecific AbEn molecule exhibited an average molecular weight corresponding to the average molecular weight of the two parental molecules. The monomeric purity was assessed via HPLC-SEC on an Agilent 1260 Infinity II LC system using an AdvanceBio SEC column, with an isocratic method of 150 mM KH_2PO_4 buffer at pH 7.3, room temperature, and detection at 280 nm, where the monomeric peak area was calculated as a percentage of the total injection to confirm over 90% exchange efficiency. The corresponding peaks for the parental and trispecific AbEn are indicated. **Abbreviations used:** LALA referred to L234A and L235A mutations; HYAL, hyaluronidase; Fc, fragment crystallizable region; CAF, cancer-associated fibroblast; AbEn, antibody-enzyme fusion; cFAE, controlled Fab-arm exchange; 2-MEA, 2-mercaptoethylamine; min, minutes; mAU, Milliabsorbance at 220 nm.

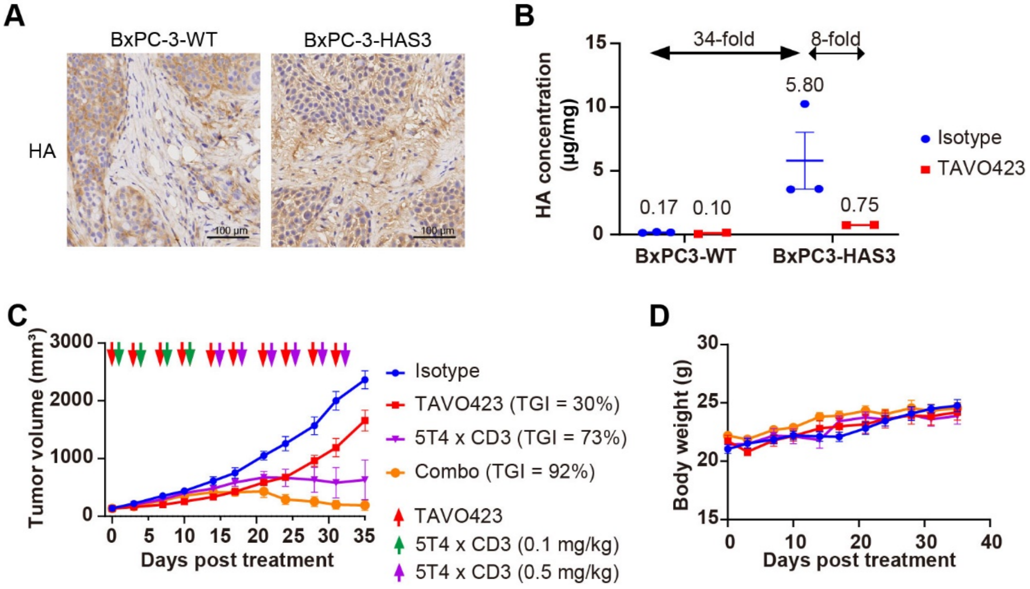

Supplementary Figure S9. TAVO423 effects on the TGI efficacy of a 5T4 × CD3 TCE in a hyaluronan-rich pancreatic cancer model. (**A**) Representative immunohistochemical staining of HA in BxPC-3 wild-type (WT) and BxPC-3-HAS3 tumor sections using an HA-binding protein. Scale bars: 100 µm. (**B**) Intratumoral HA concentration measured by ELISA from BxPC-3-WT and BxPC-3-HAS3 tumors 24 hours post-treatment. The HA concentration in µg/mg units was provided for each group and data represented the mean ± SEM values (n = 2–3 mice per group). (**C**) Tumor growth curves of BxPC-3-HAS3 xenografts treated with isotype control (blue), TAVO423 (5 mg/kg on Days 0, 3, 7, 10, 14, 17, 21, 24, 28, 31; red), 5T4×CD3 TCE (0.1 mg/kg on Days 1, 4, 8, and 11; 0.5 mg/kg on Days 15, 18, 22, 25, 29, and 32; purple), or the combination of TAVO423 and 5T4×CD3 TCE (orange). All treatments were administered twice weekly (n = 8 mice per group). (D) Body weight changes throughout the treatment period, indicating good tolerability across all regimens. Data represented mean ± SEM (n = 8 per group) values. **Abbreviations used**: WT, wild-type; TGI, tumor growth inhibition; TCE, T-cell engager; HA, hyaluronan; HAS3, hyaluronan synthase 3; mg/kg, milligram per kilogram

Supplementary Table S1. Hyaluronidase enzyme activity of the HYAL-Fc, HYAL-His, and EGFR × HYAL AbEn

Hyaluronidase	Activity[a] (U/mg)	Activity[a] (U/nM)
HYAL-Fc	41,310 ± 871	6,352 ± 134
HYAL-His	111,101 ± 1,542	5,863 ± 81
EGFR x HYAL	13,670 ± 566	2,047 ± 85

[a], Enzyme activity values were measured using a 45-minute turbidimetric assay. Data presented were the mean ± SEM values of at least three independent experiments.

Abbreviations used: U, units activity; HYAL, hyaluronidase; EGFR, epidermal growth factor receptor; SEM, standard error of the mean; Fc, fragment crystallizable; His, histidine; AbEn, antibody-enzyme fusion.

Supplementary Table S2. Binding of EGFR x HYAL AbEn to the HCC827 cell line

Molecules	EC_{50} ± SEM (nM)	E_{max} ± SEM (gMFI)
EGFR x HYAL	7.18 ± 0.3875	7.26 x 10^5 ± 14526
EGFR x Null	4.99 ± 0.2184	6.26 x 10^5 ± 10090
HYAL-Fc	NB	NB

Abbreviations used: NB, no binding; EC_{50}, median effect concentration; E_{max}, maximal effect; gMFI, geometrical mean fluorescent intensity; SEM, standard error of the mean.

Supplementary Table S3. Target Profile Screening of CDX models with HA-high stroma content

Cancer type	Cell line	FAP	LRRC15	HA
Breast cancer	MDA-MB-231	-	+	-
	MDA-MB-468	+	+	-
	HCC-70	+	+	-
Colorectal cancer	RKO	+	+	+
Lung cancer	A549	/	+	+
	H1975	+	+	-
	HCC827	+	+	+
	H1048	-	-	-
Pancreatic cancer	BxPC-3	+	+	-
	AsPC-1	/	/	+

**Gastric cancer**	**N87**	/	/	+
	**MKN45**	-	+	-
	**SNU-5**	+	+	-
**Prostate cancer**	**PC3**	-	+	-

"+": positive staining; "-": negative staining; "/": not detected.

Supplementary Table S4. Binding properties of anti-FAP x anti-LRRC15 bispecific antibodies

**Antibodies**	**Antigen/Cell lines**	**EC_{50} (nM)**	**95% CI**	**Number of replicates**
FAP x LRRC15	Human FAP	0.07988	0.07086 to 0.09006	3
	Mouse FAP	0.05930	0.04745 to 0.07405	3
	Human LRRC15	0.1326	0.1061 to 0.1656	3
	Mouse LRRC15	13.58	9.062 to 21.82	3
FAP	U87-MG	12.68	3.795 to 127.1	3
LRRC15		0.8885	0.7094 to 1.114	3
FAP x LRRC15		0.2645	0.1952 to 0.3571	3

Abbreviations used: EC_{50}, median effect concentration; CI, confidence interval.

Supplementary Table S5. Tumor growth inhibition values of TAVO423 combined with various antitumor drugs

**Figure Reference**	**Treatment**	**TGI**
Figure 5A	TAVO423	28%
	5-FU	14%
	Combo	49%
Figure 5B	TAVO423	28%
	Anti-PD-1	14%
	Combo	56%
Figure 5E	TAVO423	28%
	PD-L1 x CD3	1%
	Combo	55%
Figure 5F	TAVO423	18%
	CD318-MMAE	21%
	Combo	57%

Abbreviations used: 5-FU, 5-Fluorouracil; TGI, tumor growth inhibition; Combo, combination.

Supplementary Table S6. Statistical analyses of immune cells populations of tumor samples via IHC staining

**Treatment**	**CD3**		**CD8**		**CD45**		**CD11b**	
	Positive Cells (%)[a]	Fold change[b]	Positive Cells (%)	Fold change	Positive Cells (%)	Fold change	Positive Cells (%)	Fold change
**Isotype**	0.9	1.0	0.9	1.0	38.1	1.0	57.8	1.0
**FAP x LRRC15 x HYAL**	1.1	1.2	1.0	1.1	45.7	1.2	66.1	1.1
**Anti-mouse PD-1**	3.5	3.8	3.0	3.3	51.8	1.4	66.4	1.1
**Combo**	8.0	8.7	5.5	6.0	68.3	1.8	77.6	1.3

[a], refers to the percentage of CD3, CD8, CD45, and CD11b positive cells in tumor samples;

[b], refers to the ratio of the percentage of positive cells normalized to the isotype control;

Abbreviations used: Combo, combination; IHC, immunohistochemistry.

Supplementary Table S7. Profiles of the drugs and antibody constructs

**Name**	**Target**	**Format**	**Referenced in**
EGFR x HYAL	EGFR, HA	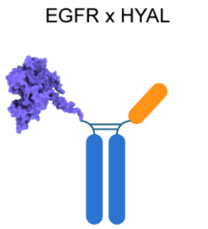	Supplementary Figure S1A; Figure 1; Figure 2
PD-L1 x HYAL	PD-L1, HA	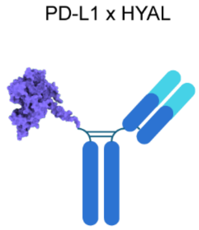	Supplementary Figure S1B; Figure 3
HAYL-Fc	HA	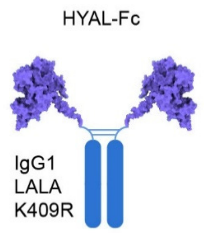	Figure 1; Figure 2
TAVO412	EGFR, cMet, VEGF	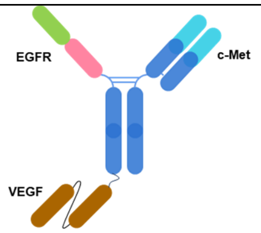	Figure 3
5-FU	Thymidylate synthase	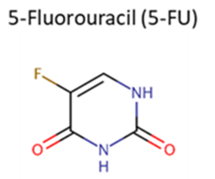	Figure 5

Anti-PD-L1 mAb	PD-L1	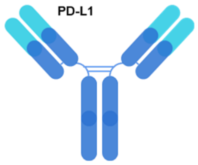	Figure 5
PD-L1 x CD3	PD-L1, CD3	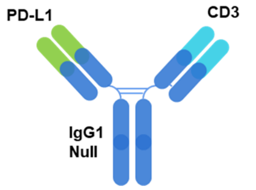	Figure 5
CD318-MMAE	CD318	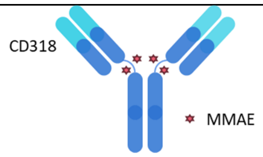	Figure 5
TAVO423	FAP, LRRC15, HA	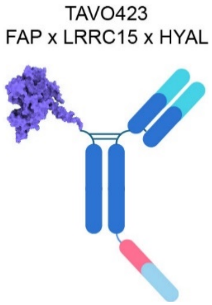	Figure 4; Figure 5; Figure 6
5T4 x CD3	5T4, CD3	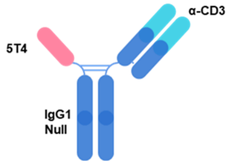	Supplementary Figure S9
Anti-mouse PD-1 mAb	PD-1	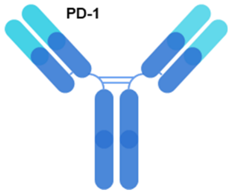	Figure 6
